# XAI-Driven Intrusion Detection for Internet of Things Networks

**DOI:** 10.3390/s26175437

**Published:** 2026-08-28

**Authors:** Awatif Alqahtani, Fatimah Alakeel, Lujain Abuhaimed

**Affiliations:** 1 Computer Science and Engineering Department, College of Applied Studies, King Saud University, Riyadh 11451, Saudi Arabia; fyalakeel@ksu.edu.sa; 2 Master’s of Cybersecurity Program, College of Computer and Information Sciences, King Saud University, Riyadh 11451, Saudi Arabia; 444203467@student.ksu.edu.sa

**Keywords:** intrusion detection systems, explainability, ensemble learning, IoT attacks, cybersecurity, CICIoT2023, TON_IoT, Edge-IIoTset, LIME, SHAP

## Abstract

Systems that can reliably detect intrusion are increasingly in demand as the scale and heterogeneity of Internet of Things (IoT) networks rise. However, when strong tabular models are employed as a benchmark, it is not clear whether the complexity of ensembling actually pays off. The aim of this work is to systematically assess the reliability and accuracy of soft-voting ensembles across three benchmark datasets (CICIoT2023, TON_IoT, and Edge-IIoTset), applying a leakage-free protocol with twice-repeated stratified 10-fold cross-validation. Ensembles of varying sizes and compositions were benchmarked against Decision Tree, KNN, Random Forest, LightGBM, XGBoost, and CatBoost and the results revealed that ensemble complexity did not lead to a consistent increase in performance. For example, SoftVote-2 increased macro-F1 over LightGBM on CICIoT2023 from 0.8506 to 0.8563, whereas LightGBM remained superior on TON_IoT and Edge-IIoTset. A leakage analysis demonstrated that resampling before data partitioning increased accuracy by around 8 percentage points and macro-F1 by 14 to 17 percentage points. To assess the trade-off between performance and complexity, training time, inference latency, memory, and model size were all evaluated, and LIME and SHAP were also assessed for explanation stability, local fidelity, and attribution agreement. XAI-guided feature selection showed that the 15 most important features retained 98.4–99.4% of the original macro-F1 and decreased inference latency by up to 38%. The results indicate that the value of ensemble complexity varies by dataset and should be weighed against its computational cost. The main contribution of this study is a leakage-aware and explainability-informed framework that can be used to judge when ensemble complexity yields genuine predictive and practical benefits for the detection of IoT intrusion.

## 1. Introduction

The Internet of Things (IoT) is a network of physical objects that are able to perceive and interact with themselves and/or the surrounding environment. These devices collect data for different purposes, facilitated by the controller and cloud computing [1]. The IoT can be utilized to connect objects; it can make access to devices, and thus their control and management, much easier, opening up a range of uses in diverse scenarios, including smart homes, smart industries, smart transportation, and smart healthcare. Automation of device control can make it easier for people to use them, increasing comfort and convenience and ultimately increasing quality of life. However, a severe drawback is that IoT devices are vulnerable to cyberattacks, which can affect the integrity of the manufacturing process and expose critical data to potential risks [2]. Examples of common attack vectors that target IoT networks and devices are denial-of-service (DoS) attacks and distributed denial-of-service (DDoS) attacks [3]. A DoS attack is a deliberate attempt to prevent authorized users from accessing a service or a network resource [3], while a DDoS attack is the deliberate use of multiple dispersed sources to launch a DoS attack. The frequency and intensity of DoS and DDoS attacks are still increasing, but on average, 28,700 attacks occur every day [3,4,5]. In light of the above, it is clear that it is crucial to develop solutions that can effectively detect and mitigate DoS and DDoS attacks. At the moment, the main security mechanisms used to protect IoT network components from cyberattacks are firewalls, intrusion prevention systems (IPSs), and intrusion detection systems (IDSs) [3]. However, the prevention of complex distributed DDoS attacks is beyond the capabilities of such mechanisms [3,6,7,8,9]. The limited adaptability of IDSs and IPSs, which traditionally rely on fixed and predefined rules to distinguish legitimate from malicious network traffic, can reduce their effectiveness against evolving and previously unseen attacks. In contrast, incorporating Artificial Intelligence (AI) techniques into IDSs and IPSs can improve how reliably and effectively they detect and prevent attacks by allowing systems to learn patterns from network traffic rather than relying solely on predefined criteria [3,7]. To the best of our knowledge, although previous studies have applied explainability techniques to the CICIoT2023, TON_IoT, and/or Edge-IIoTset datasets, our study is the first to integrate predictive performance with model interpretability within a unified framework. Specifically, we develop an ensemble comprising three machine learning (ML) models and combine them through a soft-voting strategy to optimize classification performance. Using a consistent evaluation protocol, we also examine when the ensemble provides an advantage over its strongest individual model and when such an advantage is not observed. To improve transparency, Local Interpretable Model-agnostic Explanations (LIME) is used to provide local explanations of the ensemble’s predictions. These local explanations are then compared with global feature-importance rankings derived from SHapley Additive exPlanations (SHAP) to evaluate the consistency of the identified influential features and provide complementary evidence regarding the reliability of the explanations. Additionally, reported accuracies for IoT intrusion detection vary widely across the literature, but the underlying tasks are not equivalent: studies differ in class granularity, in the proportions of the datasets used, in the class distribution of the evaluation data and, as we quantify in Section 5.1, in whether resampling is applied before or after the train/test split. To address these gaps, this study offers a systematic assessment of the value of ensemble complexity in IoT intrusion detection, focusing on whether combining classifiers confers significant advantages compared with strong single tabular learners. The assessment process looked at predictive performance, reliability, computational and deployment costs, and the reliability and practical utility of model explanations. The main contributions of this study are as follows:

- **Quantify the marginal value of ensemble complexity** by using different ensemble sizes, compositions, and resampling strategies in the three IoT datasets, and then making comparisons between ensembles and strong single learners such as LightGBM. This establishes when it is advantageous to employ additional classifiers and when it is not (Section 5.3, Section 5.4 and Section 5.7).
- **Establish a leakage-free evaluation protocol** and quantify the effect of resampling before data partitioning. The results of this showed how evaluation leakage could inflate the reported performance in accuracy and in macro-F1 (Section 5.1).
- **Quantify the trade-off between complexity and performance** using training time, inference latency, memory, and model size. This establishes whether the increased processing and deployment costs associated with ensemble complexity are justified by improvements in prediction accuracy (Section 5.5).
- **Evaluate explanation reliability and practical utility** using LIME stability, surrogate fidelity, SHAP agreement, and XAI-guided feature selection. This demonstrates whether explanations can enable useful feature reduction and deployment optimization, as well as the required level of reliability for interpretation (Section 5.6).

This paper is structured as follows: Section 2 presents related studies, and Section 3 sheds light on the theoretical background of the employed algorithms and techniques. Our key contribution is discussed in Section 4, which presents our proposed model and the study’s methodology. Then, Section 5 presents the results, and Section 6 discusses them. We conclude the study and highlight possible future research directions in Section 7.

## 2. Related Work

The CICIOT2023 dataset has become a benchmark for evaluating IDSs in IoT networks because it captures several IoT attacks not represented in other IoT security datasets [10]. Several studies have combined explainable artificial intelligence (XAI) techniques with machine and deep learning approaches to enrich the interpretability of the resulting IDS models. This section discusses studies that have utilized CICIoT2023, including a number that combined their model results using ensemble learning techniques, and some that applied explainability to make IDS models more transparent and understandable. The work by Adewole et al. [11] developed an IDS framework for IoT using an ensemble learning approach with five ML algorithms: Random Forest, AdaBoost, XGBoost, LightGBM, and CatBoost. Testing the framework on two public datasets, XGBoost outperformed other algorithms. The authors employed rule induction for enhanced model explainability, where the induced rules helped users to understand the IDS framework’s decisions. However, they did not apply SHAP as an explainable approach, which we aim to do in our study to improve the transparency of IoT attack categorization. Focusing on detecting DDoS, the work by Saiyed et al. [12] employed an attention-based ensemble learning approach using deep learning methods, specifically Convolutional Neural Network (CNN) and Long Short-Term Memory (LSTM), to build an IDS framework, namely ADEPT. The framework was tested on several datasets and the authors employed SHAP to enhance model transparency and interpretability. Although the authors tested their work using different datasets and applied SHAP, the overall accuracy of the results was 90% for detecting DDoS attacks for both low and high volumes. There are several studies [13,14,15] that apply explainability without applying the ensemble learning approach. For example, Tewari et al. [16] built an IDS that combined a CNN with Bidirectional Long Short-Term Memory (BiLSTM) algorithms, yielding 85.56% accuracy. The authors used SHAP and LIME to explain the model, and the accuracy improved by 4% with SHAP-based feature selection. Additionally, Hajjouz and Avksentieva [13] built an IDS framework using CatBoost, achieving a result of 99.96% accuracy. The authors employed SHAP, which provides both global and local interpretability, highlighting feature importance across different attack subtypes. Other work, such as [13,16], applied ML but no ensemble techniques. In order to enhance IoT security, the authors of the [17] study suggested a hybrid architecture of Neural Network (NN) models called HRSNN, which combines Recurrent-NN (RNN) and Spiking-NN (SNN). The hybrid model improved the accuracy and robustness of detecting anomalies in IoT networks by combining the temporal adaptability of SNNs with the spatial feature-learning capabilities of RNNs. They tested it using both the CIC-IoT23 and TON_IoT datasets, and the suggested HRSNN method outperformed the existing deep learning (DL) models. The model achieved an accuracy rate of 99.5% and 98.75% on the “CICIoT2023” and the “TON_IoT” datasets, respectively. However, despite its great accuracy, this work did not use an ensemble strategy or explainability approach, both of which are employed in the current study.

By combining statistical feature selection (ANOVA) with SHAP and LIME, ref. [14] presents an effective and comprehensible XAI-based IDS framework that improves interpretability while maintaining high detection performance and computational efficiency, thereby mitigating the “blackbox” limitations of traditional AI-IDS. The two-stage approach validates the accuracy of the interpretations by reducing the feature space and enforcing consistency between SHAP and LIME local explanations. Strong attack-detection capabilities across a variety of networking environments and attack scenarios are demonstrated by a thorough evaluation of the CIC-DDoS2019, CICIoT2023, and 5G PFCP datasets. With the reduced feature set, the framework sustains the F1 score while speeding up the generation of LIME explanations by up to 87%. While we are looking into using an ensemble approach to improve the accuracy of intrusion detection attacks, the authors used an XGBoost model.

To increase the transparency and resilience of DL-based IDSs in IIoT networks, the work described in [18] provides an explainable ensemble DL-based IDS. For experts in charge of preserving IIoT network security and creating more cyber-resilient systems, the framework uses SHAP and LIME techniques to clarify the choices made by DL-based IDSs. The effectiveness of the proposed framework was assessed using the ToN_IoT dataset. The extreme learning machines (ELM) model was used as a baseline intrusion detection system and contrasted with other models. Experiments demonstrate how well ensemble learning works to enhance outcomes. However, this work, even if it applied an ensemble approach as a model and applied LIME and SHAP for explainability purposes, was tested using the ToN_IoT dataset.

In order to improve threat detection and response, the work in [15] introduced a revolutionary end-to-end framework for IoT security that combines Explainable AI (XAI) with Large Language Models (LLMs). This approach guarantees the interpretability of ML model predictions by combining an intelligent LLM Agent with XAI techniques such as SHAP and LIME. The efficacy of the suggested methodology in identifying and reducing threats was confirmed by empirical tests conducted on the CIC-IOT-2023 dataset, demonstrating its versatility and potential for protecting IoT networks. The authors used the CIC-IOT-2023 dataset to train a random forest classifier in order to evaluate the effectiveness of the interpretation module of the suggested framework. During testing, the final model showed excellent performance, with an accuracy of about 99.97%. It did not, however, use an ensemble approach.

A major security issue in IoT contexts is addressed by the work provided in [19], which presents a DL methodology for identifying and categorizing DDoS threats. Two benchmarking datasets of DDoS attacks were used to assess the accuracy and time complexity of DL backbones using an efficient deep transfer learning approach. The study carried out extensive binary and multiclass trials, each with different degrees of complexity in identifying attack types and illustrating real-world scenarios, by utilizing multiple deep architectures. Furthermore, an XAI technique was used to clarify the role that extracted features play in attack detection. The XAI bidirectional long short-term memory (XAI-BiLSTM) model achieved a 99.39% recall in the trial, proving the efficacy of the suggested approach. It did not, however, use an ensemble approach. The results of Abid et al. [20] are an example of the strong performance that can be delivered by strong single tabular learners. The authors evaluated gradient-boosting classifiers on CICIoT2023 and reported a macro-F1 of 0.8903 for XGBoost in the 8-class classification setting. The potential of ensemble-based models has also been demonstrated by [21], who combined several classifiers with hybrid feature selection and explainability to develop a robust IoT intrusion-detection framework that achieved a macro-F1 of 0.7379 on CICIoT2023 [21]. Shaikhanova et al. [22] evaluated several machine-learning classifiers on the multiclass TON_IoT dataset and reported a macro-F1 of 0.9694 for LightGBM, further proving that extremely competitive intrusion-detection performance can be attained by a powerful single tabular learner.

As far as we are aware, our study differs from previous work because it combines three ML models to form a performance-optimized ensemble using a soft-voting approach. While other research has utilized the CICIoT2023, TON_IoT, and/or Edge-IIoTset datasets with explainability approaches, it is rare to examine detection performance and interpretability together in a unified framework. Furthermore, this study assesses the circumstances under which the ensemble improves upon its strongest individual member, using a standard experimental technique. The decision-making process of the proposed model is explained through LIME, making its predictions more transparent and understandable. Finally, the consistency and reliability of the significant features identified by the model are evaluated by comparing the LIME explanations with the global feature-importance rankings obtained from SHAP.

Table 1 presents a summary of the reviewed studies, underscoring the obvious gap in the existing literature. There is a lack of research assessing intrusion detection across the three datasets used here while also employing ensemble learning, a rigorous 10-fold cross-validation protocol repeated twice (10-fold CV × 2), and a leakage-free evaluation framework. Furthermore, researchers have not compared local explanations based on LIME with the high-importance features identified via SHAP. In an attempt to fill these gaps, this study combines ensemble learning with leakage-free evaluation and quantitatively evaluates LIME explanations against the features identified by SHAP. This integrated framework constitutes the primary focus and contribution of the present research.

## 3. Theoretical Background

This section presents a summary of the AI technologies that were considered in this research. The creation, enhancement, and interpretation of analytical models can be greatly aided by the use of gradient boosting, decision trees (DTs), k-Nearest Neighbor (kNN), ensemble algorithms and techniques, and LIME. Each of these techniques contributes to the development of precise, quickly interpreted models that can learn quickly, thereby addressing various problems related to model assessment, training, and explanation. For this reason, the DT and kNN algorithms are used in our study to determine the security risks in IoT-related datasets. In addition, LIME approaches are used to evaluate the results of the black-box models.

### 3.1. Decision Tree

The DT is a popular classification algorithm for data mining. It can handle massive volumes of data efficiently and be used to classify newly acquired data and information based on training sets and class labels, and to draw conclusions about categorical class names [23]. This supervised learning algorithm gains its name from the fact that by repeatedly dividing the data into subsets based on the input feature values, a tree structure is created [24]. A DT is made up of leaf nodes that reflect the class value and decision nodes that represent tests on one or more characteristics [24,25]. Each route from the root node to the leaf node is a rule. The split condition implemented at every node should yield subsets that are homogeneous, that is, they consist of records that share the same class label. When constructing the decision tree, the objective at each node is to choose split conditions that effectively separate the dataset into homogeneous subsets. Ref. [26] introduced the “goodness of split criterion”, which is based on the concept of impurity. The level of impurity is quantitatively assessed for each split condition, and the split condition with the lowest impurity value is selected [26]. The impurity value of a divided condition is measured in DT using a range of indices, such as information gain and the GINI index [26]. This section provides a brief discussion of the theoretical concepts related to information gain and the GINI index. According to [26], Raileanu and Stoffel [27] conducted a theoretical analysis comparing the GINI index and Information gain:

Let S be a learning sample, $S=\left\{\left(y_{1},{\mathrm{cl}}_{1}\right),\left(y_{2},{\mathrm{cl}}_{2}\right)\ldots \left(y_{i},{\mathrm{cl}}_{j}\right)\right\}$; Where $y_{1},y_{2}\ldots y_{i}$ is a measurement vector and $cl_{1},cl_{2}\ldots cl_{j}$ are class labels. $y_{i}$ can be presented as a vector of input variables. Then, the split conditions are built based on one of these variables. If $p_{i}$ is the probability of any given tuple that belongs to ${\mathrm{cl}}_{i}$ class, $p_{i}$ can be calculated as

(1) $$p_{i}=\frac{cl_{i}}{S}$$

Relationship to random forests. The base learner used in this work is a bagging ensemble of decision trees, which is related to but distinct from a random forest: bagging resamples the training instances only, whereas a random forest additionally samples a random subset of features at each split. This distinction is stated here to avoid confusion: to make the comparison explicit, Random Forest is evaluated as a separate baseline in Section 5.1.

#### 3.1.1. Entropy

Entropy estimates the degree of disorder or unpredictability in a dataset [26,28]. If the subsets of a dataset show homogeneity, it indicates that the dataset lacks impurity or randomness. If a single label class categorizes all the observations within subsets, the dataset’s entropy would be 0. By adding the probabilities of each label and the logarithm of that label’s probability, we can compute entropy.

(2) $$\begin{array}{cc} \mathrm{Entropy}(L)= & \;\langle C_{1}|L\rangle \log_{2}\langle C_{1}|L\rangle \\ \; & +\langle C_{2}|L\rangle \log_{2}\langle C_{2}|L\rangle +\ldots \\ \; & +\langle C_{j}|L\rangle \log_{2}\langle C_{j}|L\rangle \end{array}$$

(3) $$\mathrm{Entropy}\left(L\right)=p_{1}\log_{2}p_{1}+p_{2}\log_{2}p_{2}+\cdot \cdot \cdot +p_{j}\log_{2}p_{j}$$

(4) $$\mathrm{Entropy}\left(L\right)=-\sum_{i=1}^{j}p_{i}\log_{2}\left(p_{i}\right)$$

In a dataset with only one class label, the value of $p_{i}$ will be 1, and  $\log_{2}\left(p_{i}\right)$ is 0. Consequently, the entropy of a homogeneous data set is 0 [29]. When the entropy is higher, there is a higher level of uncertainty, impurity, or mixing [30].

#### 3.1.2. Information Gain

Information gain is derived from entropy since, given a specific feature, it is computed based on the difference between the entropy of a class and the conditional entropy of the class. Information gain assesses the reduction in uncertainty that results when the dataset is partitioned according to a particular characteristic, such as f [31]. An increasing information gain value suggests that the feature f is increasingly helpful for categorization. The best feature to take into account for a split is the one with the highest information gain.

Let V be the number of distinct values for a feature f, and the subset of *L* with f=v is denoted as $\left|L^{v}\right|$, so when splitting *L* using a feature *f*, the measurement of information gain is formulated as follows [29]:

$$IG\left(L,f\right)=\mathrm{Entropy}\left(L\right)-\sum_{v=1}^{V}\frac{\left|L^{V}\right|}{\left|L\right|}\left(\mathrm{Entropy}\;\left(L^{V}\right)\right)$$

#### 3.1.3. GINI Index

GINI index determines the purity of a specific class. Once a class has been divided along a specific attribute, the GINI index calculates the purity of that class. The sets that result from the split are purer when the best split is used. If *L* is a dataset with *j* different class labels, GINI is defined as [32]:

(5) $$p_{i}=\frac{C_{i}}{L}$$

$p_{i}$ represents the relative frequency of class *i* in *L*. When the dataset is divided based on attribute *A*, resulting in two subsets L1 and L2 with sizes N1 and N2 correspondingly, the GINI index is computed as follows:

(6) $${\mathrm{GINI}}_{A}\left(L\right)=\frac{N_{1}}{N}\mathrm{GINI}\left(L_{1}\right)+\frac{N_{2}}{N}\mathrm{GINI}\left(L_{2}\right)$$

Reduction in impurity is calculated as

(7) $$\Delta \mathrm{GINI}\left(A\right)=\mathrm{GINI}\left(L\right)-{\mathrm{GINI}}_{A}\left(L\right)$$

### 3.2. k-Nearest Neighbour (kNN)

Fix and Hodges [33] introduced the kNN algorithm, a supervised, non-parametric learning method, in their groundbreaking research. The input for this method is the k training samples from a given dataset that are closest to the object of interest [34]. The algorithm’s output for classification problems is assigning an object to a class determined by the majority class membership among its k nearest neighbors. Let us examine a specific set of observations D:

(8) $$D=\left\{\left(x_{1},y_{1}\right),\ldots ,\left(x_{N},y_{N}\right)\right\}\;j=1,\ldots ,N$$

where $x_{j}$ is the feature vector and $y_{j}$ is the associated category/class label of the *j*-th example. The $\left(x_{j},y_{j}\right)$ is assumed to be j.j.d. from some distribution of unknown form *P* of (x,y) on $R^{d}\times \left\{\omega_{1},\ldots ,\omega_{H}\right\}$, where *H* represents the number of categories, and the main purpose is to design a mapping function $\phi_{n}:R^{d}\to \left\{\omega_{1},\ldots ,\omega_{H}\right\}$ to map a feature vector *x* into its possible category from $\left\{\omega_{1},\ldots ,\omega_{H}\right\}$.

The classifier’s performance is assessed using Equation (2)’s probability error [35]:

(9) $$K\left(\phi_{n}\right)=P\left\{\left(x,y\right):\phi_{n}\left(x\right)\neq y\right\}$$

where *K* is the cost function and the classifying function is $\phi_{n}$. Equation (3) illustrates how to use the Bayes decision rule to find the optimal decision rule $\phi^{*}$ that minimizes the likelihood of error when the underlying distribution is known:

(10) $$\phi^{*}\left(x\right)=\mathrm{argmax}P\left(y|x\right)$$

It can be shown that for each given point, denoted as *x*, the likelihood that its nearest neighbor, denoted as $x^{\prime }$, belongs to class $\omega_{j}$ approaches the related a posteriori probability $P\left(\omega_{j}|x^{\prime }\right)$ as the number of reference observations tends towards infinity. Cover and Hart [36] showed that, subject to certain continuity assumptions on the underlying distribution, for a multi-class kNN classifier, the asymptotic probability of error $L_{NN}$ can be bounded by the parameters specified in Equation (4).

(11) $$L^{*}\leq L_{NN}\leq L^{*}\left(2-\frac{H}{H-1}L^{*}\right)$$

### 3.3. Gradient Boosting

Gradient Boosting is an advanced method in ML for model enhancement with the help of many weak predictors, normally decision trees, to create a strong model. The process is repeated—each new model tries to address the mistakes made by the previous models by minimizing a loss function with the help of gradient descent [37].

Friedman (2001) [37] introduced the Gradient Boosting Machine (GBM), which is one of the best-regarded uses of Gradient Boosting, and it has proven very successful in several practical applications such as risk modeling or anomaly detection. Additionally, some of its recent evolutions are well-known algorithms such as XGBoost, LightGBM, and CatBoost, which aim to improve speed, accuracy and scalability on big data sets (Ke et al. 2017) [38].

The basic idea in Gradient Boosting is to fit a model with the least squared error with respect to the given loss function: L(y, F(x)), where y represents the true labels and F(x) is the model’s prediction:

(12) $$F_{m}\left(x\right)=F_{m-1}\left(x\right)+\nu \cdot h_{m}\left(x\right)$$

Here, the new model is established following the addition of the m(th) weak learner $h_{m}\left(x\right)$ such that the function of this new model is denoted by $F_{m}\left(x\right)$, whereas the learning rate that supersedes the products by each learner’s share is represented by *v*.

For regression problems, the most common loss function is Mean Squared Error (MSE):

(13) $$L\left(y,F\left(x\right)\right)=\frac{1}{2}{\left(y-F\left(x\right)\right)}^{2}$$

During each iteration, the gradient of the loss function is calculated for the current prediction

(14) $$g_{m,i}=\frac{\partial L(y_{i},F_{m-1}\left(x_{i}\right))}{\partial F_{m-1}\left(x_{i}\right)}$$

The new model $h_{m}\left(x\right)$ is then trained to fit the negative gradient:

(15) $$h_{m}\left(x\right)=\arg\min_{h}\sum_{i=1}^{n}{\left[-g_{m,i}-h\left(x_{i}\right)\right]}^{2}$$

In conclusion, the update of the general model is completed by integrating this fresh weak learning agent:

(16) $$F_{m}\left(x\right)=F_{m-1}\left(x\right)+\nu \cdot h_{m}\left(x\right)$$

### 3.4. Local Interpretable Model-Agnostic Explanations (LIME)

By determining the classifiers’ uncertainty within the space of a single instance, LIME enables the creation of readily interpretable models that emulate the behavior of complex models in the proximity of a particular instance. This technique is valuable if a model’s decision-making process needs to be explained. For a provided instance *x*, LIME generates a number of samples and fits a simple linear model $g(x^{\prime })$ locally around *x* [39]:

(17) $$g\left(x^{\prime }\right)=\arg\min_{g\in G}\left[L\left(f,g,\pi_{x}\right)+\Omega \left(g\right)\right]$$

where:

- $L(f,g,\pi_{x})$ measures the falseness of the approximation *g* compared with the actual model *f*,
- $\pi_{x}$ is another measure in the distance metric that weights the perturbed samples using the distance *x*,
- Ω(g) measures the complexity for the interpretable model *g*.

LIME is able to simplify the explanation of complex models by providing an explanation of the model’s decision-making process close to the specific instance.

Two properties of LIME matter for intrusion detection and they motivate the evaluation in Section 5.5. First, LIME uses random sampling, so explaining the same instance twice can give different results; this instability is a known limitation of LIME. Second, the simple model g only approximates f near one instance, so the quality of the approximation changes from one instance to another and from one class to another. A single explanation figure cannot show either of these properties, so we measure both in Section 5.5.

### 3.5. Ensemble Techniques

Ensemble methods are among the fundamental concepts of ML, as they integrate multiple models in order to produce a more effective overall model. The rationale for this is that specific models may have certain deficiencies that can be compensated for by the ensemble of the models to lower variance and bias and increase predictive accuracy.

Techniques like this particularly succeed in the determination of variance, bias, or the improvement of predictions using a mixture of models. For instance, random forests use bagging to create numerous decision trees, while boosting methods such as AdaBoost aim to correct the mistakes made by previous models. Moreover, these ensemble methods have been widely employed in practical situations such as financial modeling and disease prediction because they can help improve model performance.

#### 3.5.1. Bagging (Bootstrap Aggregating)

In bagging, each model in the ensemble is trained on a different data subset, and then their predictions are averaged for regression or voted on for classification.

(18) $$\hat{y}=\frac{1}{M}\sum_{m=1}^{M}h_{m}\left(x\right)$$

where *M* represents the total number of models in the ensemble, and $h_{m}\left(x\right)$ is the prediction output of the *m*-th model.

#### 3.5.2. Boosting

The sequential training of models is what happens during boosting, whereby each novel model concentrates on correcting the errors of prior models. The ultimate forecast aggregates the predictions of all the models, taking their weights into consideration.

(19) $$\hat{y}=\sum_{m=1}^{M}\alpha_{m}\cdot h_{m}\left(x\right)$$

where $\alpha_{m}$ is the weight assigned to the *m*-th model, determined based on its performance.

Due to their ability to improve model robustness and accuracy, these ensemble strategies have become popular among other domains including finance and health care.

#### 3.5.3. Voting

The voting ensemble is mostly used for classification purposes as it learns from two or more learning models that use the same dataset. Additionally, it is an ML model that reaches the predicted class based on the highest probability of more than one of the independent models [40]. There are two types of voting techniques: hard voting and soft voting. In hard voting, the model predicts the output class based on the class that has the highest number of votes from each of the classifiers. For example, if there are three classifiers that predict the output class as (A, B, B), which means that the majority predicted B, the output class is B. In soft voting, the output class is predicted by averaging the probability assigned to each class by the classifiers, and then the output class is the one with the highest averaged probability. Assume that there are three models, and the prediction probability for class A = (0.47, 0.30, 0.53) and for class B = (0.32, 0.40, 0.20). The average for class A is 0.4333, and for class B, it is 0.3067. Thus, the output class is A, as it obtained the highest probability averaged by each classifier [40].

## 4. Methodology

**Figure 1:**
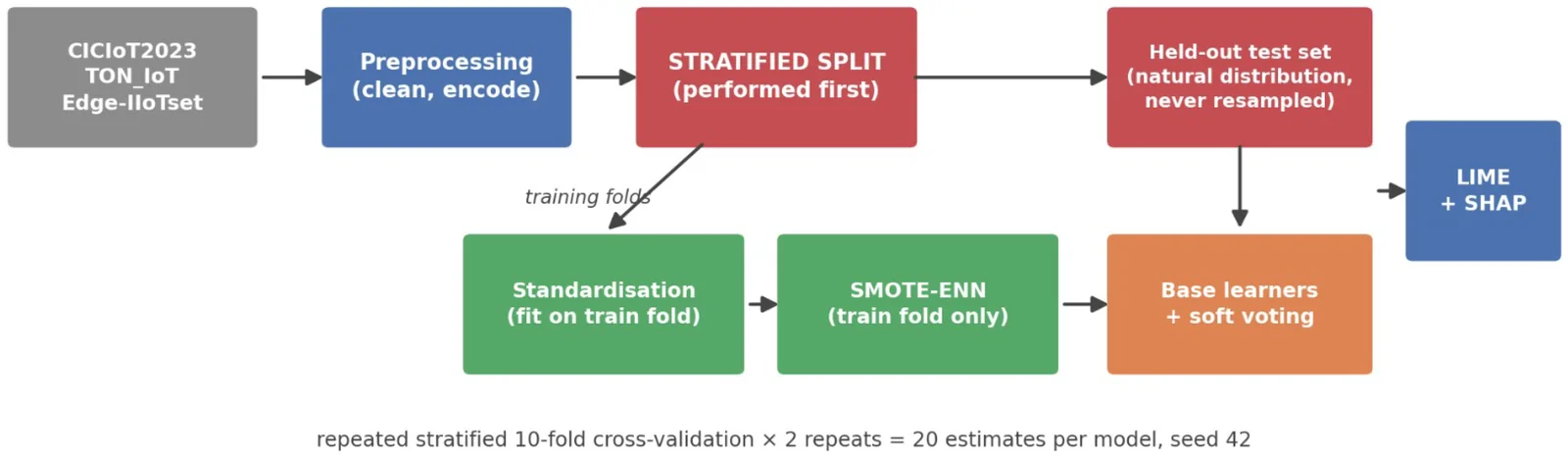
Proposed framework.

This section outlines our suggested method for employing ML algorithms and XAI techniques (see Figure 1). It comprises the following main stages: data preprocessing, handling data imbalance, optimizing algorithm parameters, training individual models with these parameters, and constructing a voting ensemble from these models. Looking at how influential the key features are, we assess the ensemble’s performance and apply the LIME framework to interpret the ensemble’s decision-making process. A stratified split is used to maintain the original class proportions when dividing the data; this ensures that the proposed model is evaluated in an unbiased manner. The SMOTE-ENN resampling process and standardization parameters are then individually fitted and applied inside each training fold, and all preprocessing operations are then carried out solely within the training data, in order to prevent the leakage of information between the training and validation data. It is important to note that the held-out test set is not used for resampling but remains untouched, retaining its natural class distribution. This ensures that the end evaluation reflects the model’s performance under the original, real-world class distribution.

### 4.1. The Utilized Dataset

In our work, we utilized the CICIoT2023 Dataset provided by [10]. The main goal of the research described in [10] was to offer a novel and extensive dataset on IoT attacks. In order to facilitate the development of security analytics applications in real-world IoT environments, they released a new and genuine IoT attack dataset. The dataset was collected using a broad topology with a large number of real IoT devices. Ref. [10] observed, documented, and collected information from 33 attacks on IoT devices, which were divided into seven different classes: Web-based attacks, Brute Force attacks, Mirai attacks, DoS, DDoS, Reconnaissance (Recon), and Spoofing attacks. These attacks were all initiated by malicious IoT devices, charged with the objective of disrupting other IoT devices.

Table 2 shows the seven categories as well as their subcategories; the rows correspond to the subclasses, and the columns represent the primary categories.

For further details such as the subcategories of each type of attack, the details of the collected data, and the structure of the produced files, please refer to [10].

The full CICIoT2023 capture is heavily attack-dominated, and a subsample is used in this study and documented here for reproducibility. The subsample comprises 1000 flows for each of the 33 fine-grained attack classes together with 33,000 benign flows, giving 66,000 rows in total, and the row indices of the source capture are retained so that the draw is reproducible. Under the eight-class grouping used throughout the paper, the composition is Benign 33,000; DDoS 12,000; Web 6000; Recon 5000; DoS 4000; Mirai 3000; Spoofing 2000 and Brute Force 1000.

**Figure 2:**
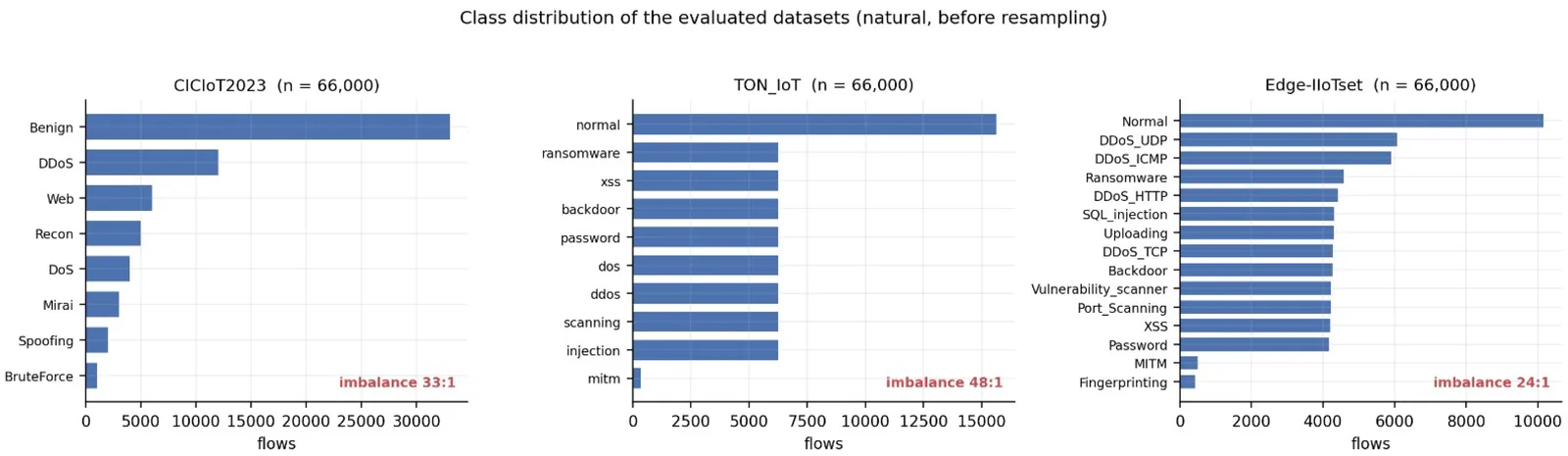
Class distributions of the three datasets [10,41,42,43] before resampling showing their natural class proportions. The imbalance ratio between the largest and smallest classes is reported for each dataset.

This composition is benign-heavy, so benign traffic is the majority class rather than a minority one. The resulting task is harder than the attack-dominated distributions used in several published studies, in which a single large flood class can carry overall accuracy, and this is one reason why our headline figures are lower than in those reports. For the same reason, we adopted macro-F1 rather than accuracy as the primary metric, since macro-F1 weights every class equally and is, therefore, sensitive to failures on the rare attack classes that matter operationally. We expanded the evaluation to two more IoT intrusion-detection datasets, TON_IoT, gathered by the Cyber Range Lab at UNSW Canberra, and Edge-IIoTset, in order to give a more thorough assessment of the suggested model and examine its generalizability beyond a single dataset. A more uniform and objective evaluation of the suggested method across various data sources was made possible by using the same preprocessing and modeling workflow for both datasets without any further fine-tuning or dataset-specific optimization. Each dataset was stratified-subsampled to 66,000 instances in order to guarantee a similar evaluation. This ensures that reported performance discrepancies are attributed to the underlying data features rather than variations in sample size. IP addresses, port numbers, timestamps, flow IDs, URIs, and DNS query strings are examples of identifier-like features that were left out because they could contain testbed-specific architecture or deployment information and are, therefore, unlikely to generalize to other settings. Low-cardinality category features, on the other hand, such as protocol, service, and connection status, were kept and one-hot encoded as they could provide useful information for differentiating between malicious and benign network activity. The related dataset references include further information about these datasets’ features, methods of collection, and original sources [41,42,43]. Table 3 lists the datasets taken into consideration in this work along with their salient features, classes, majority-class distributions, and cases. The class distributions of the three datasets prior to resampling are shown in Figure 2, emphasizing the significant class imbalance found in their natural distributions.

### 4.2. Data Preprocessing

During the preprocessing stage, we addressed key tasks such as handling class imbalance, standardizing features, and encoding data.

#### 4.2.1. Handling Class Imbalance

Resampling was applied only to the training partition of each cross-validation fold, as described in Section 4.3, and the data were never used for evaluation. As shown in Figure 2, the three datasets exhibited substantial class imbalance in their natural distributions before resampling, with imbalance ratios of 33:1 for CICIoT2023, 48:1 for TON_IoT, and 24:1 for Edge-IIoTset. The class distributions after resampling within a training fold are presented in Section 5, illustrating the effect of the resampling procedure.

#### 4.2.2. Feature Standardization

**Figure 3:**
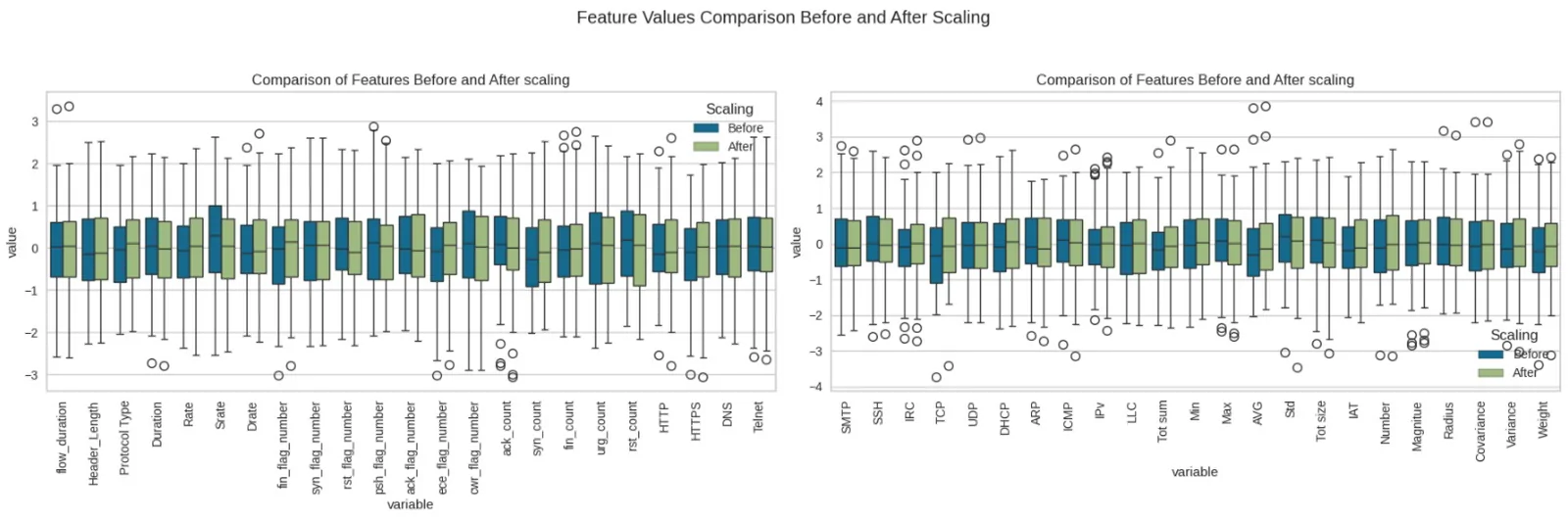
Feature Scaling.

We standardized the feature values across the dataset to ensure consistent data scale and distribution, which are crucial for the performance of many ML algorithms. This standardization was performed using the Z-score normalization method implemented by the StandardScaler from scikit-learn [44], as per the formula:

(20) $$X_{\mathrm{new}}=\frac{X_{i}-X_{\mathrm{mean}}}{\mathrm{Standard}\;\mathrm{Deviation}}$$

where $X_{\mathrm{new}}$ represents the scaled value. We applied this scaling using fit_transform on the resampled training data ($X_{\mathrm{train}\text{\_}\mathrm{resampled}}$) and transform on the test data ($X_{\mathrm{test}}$), which effectively prevented data leakage and ensured uniformity in the input data for subsequent modeling. Figure 3 shows the features before and after scaling.

The features were first standardized and resampling was applied afterwards. This sequence was used because SMOTE creates synthetic samples using distances between neighbors, and on unscaled data these distances are dominated by large-value features such as Header_Length and IAT, which bias the synthetic samples. In order to prevent a synthetic sample reaching the evaluation data, both steps were only fitted inside each training fold. The consequences of not following these steps are described in Section 5.1.

### 4.3. Data Splitting

The data were first divided into a development set and an unaltered test set (80/20, stratified) in order to assess the models’ performance. The test set data preserved the natural class distribution and were not resampled but were set aside for McNemar’s test and the explanation analysis, which is detailed in Section 5.6. We carried out two rounds of stratified 10-fold cross-validation, which gave 20 estimates per model. No data from a validation fold could influence standardization and resampling, as these were implemented as steps of a single pipeline object that was cloned and refitted on the training partition of each fold. The random seed was set as 42 for the split, the folds and all the models. The mean ± standard deviation over the 20-fold is utilized to report all of the results. We report macro-averaged precision, macro one-vs-rest AUC, and the false-positive rate on benign traffic, in addition to recall, precision, accuracy, and F1. Macro-F1 is regarded as the main metric (see Section 4.1) and we report the benign false-positive rate because it determines analyst alert load in a deployed system.

### 4.4. Ensemble Classifier

The proposed approach utilizes an ensemble method that includes Bagging Decision Trees, k-nearest Neighbors (kNN), and gradient-boosting algorithms.

#### 4.4.1. Bagging Decision Tree

The first base learner implemented was the Bagging Classifier with Decision Trees, with 100 estimators. In this approach, an ensemble of four Decision Trees was trained using the Bagging technique, with the Gini Index serving as the default criterion for splitting nodes. The purpose of the Bagging ensemble is to aggregate predictions from multiple decision trees to reduce variance and enhance model stability. The model was also trained using scaled and resampled data to address class imbalance.

#### 4.4.2. k-Nearest Neighbour (kNN)

The second base learner implemented was the kNN classifier with 7 neighbors. In this approach, classification is performed by assigning each test instance to the most common class in the feature space, within its k nearest neighbors. The kNN algorithm operates non-parametrically, meaning it does not assume any specific distribution of the data. The purpose of using kNN is to leverage the proximity-based relationships in the data, allowing it to effectively classify instances based on the majority vote among neighboring data points. Similar to the first learner, the kNN model was trained on scaled and resampled data to handle class imbalance.

#### 4.4.3. Gradient Boosting

The third base learner implemented was the gradient boosting classifier. In this approach, an ensemble of decision trees is built sequentially, where each tree is trained to correct the errors of the previous ones. 80% of the data was used for training each tree (subsample = 0.8), and 10% of the data was reserved for validation to enable early stopping if no improvement was observed after 10 iterations. The Gradient Boosting model was trained on scaled and resampled data to handle class imbalance, similar to the other base learners.

#### 4.4.4. Voting Classifier for Ensemble Learning

In our final model, we implemented a voting classifier to aggregate the predictions of multiple base learners, thereby leveraging their individual strengths. The Voting Classifier combined three previously trained models: the Bagging Classifier with Decision Trees, the k-Nearest Neighbors (kNN) classifier, and the Gradient Boosting Classifier. The voting strategy was soft voting, which takes into account the predicted probabilities of each model, thus giving more weight to models with higher confidence in their predictions.

Member selection and baselines. Soft voting is expected to help when members make weakly correlated errors: a variance-reducing bagged tree, a distance-based lazy learner, or a bias-reducing boosted model tend to fail in different regions of the feature space. This is the rationale for the original member selection. To test whether the condition holds in practice, and to address the question of why more recent gradient-boosting implementations were not considered, we additionally evaluated Random Forest, LightGBM, XGBoost and CatBoost under an identical protocol, and we report on the soft-voting ensembles of two, three and four members in Section 5.3.

The complete set of models considered in the study, together with their respective settings and ensemble configurations, is summarized in Table 4.

## 5. Results

The experimental evaluation of the suggested framework is presented in this section, covering the evaluation metrics, preprocessing order, detection performance, ablation studies, computational cost and deployment considerations, and model explainability using LIME.

### 5.1. Evaluation Metrics

The algorithms’ accuracy, precision, recall, and F1-score are used to measure their performance. True Positive (TP), True Negative (TN), False Positive (FP), and False Negative (FN) are examples of ground truth values that serve as the foundation for these metrics. They provide information on how well the model performs and, therefore, provide crucial feedback that helps to build and improve an IDS for IoT networks with the best possible functionality. The metrics are evaluated by counting the true positives, true negatives, false positives, and false negatives.

Based on the above metrics, evaluation indicators are calculated as follows:

**1. Accuracy**

(21) $$\mathrm{Accuracy}=\frac{TP+TN}{TP+TN+FP+FN}$$

**2. Precision**

(22) $$\mathrm{Precision}=\frac{TP}{TP+FP}$$

**3. Recall**

(23) $$\mathrm{Recall}=\frac{TP}{TP+FN}$$

**4. F1-score**

(24) $$F1\text{-}\mathrm{score}=\frac{2\cdot \mathrm{Recall}\cdot \mathrm{Precision}}{\mathrm{Recall}+\mathrm{Precision}}$$

**Macro-averaged** precision, recall and F1 are calculated by computing each metric per class and averaging over classes without weighting by class frequency. Resampling was carried out in two ways: with the full dataset prior to splitting, and separately within each training fold, using the same data and folds in each case. These two methods were then compared. To ensure that the findings were not attributed to one particular algorithm, three different classifiers were tested, and a paired Wilcoxon signed-rank test across folds was employed to assess whether the two approaches differed significantly.

### 5.2. Leakage-Free Evaluation

**Figure 4:**
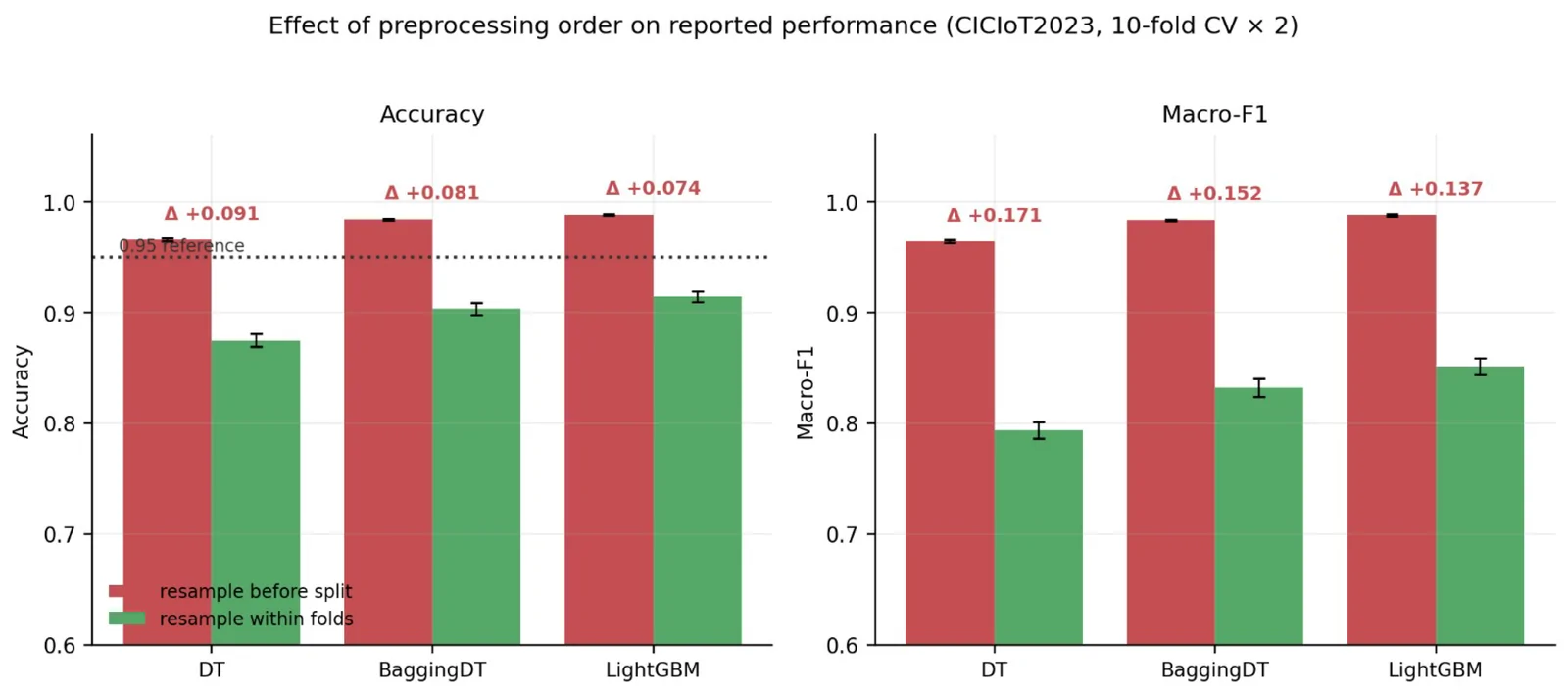
Effect of preprocessing order on reported performance. The results of applying resampling before the split are presented in red bars, and the results for resampling within each training fold are shown by green bars. The dotted line marks an accuracy of 0.95 for reference.

Table 5 presents our findings on how the results were influenced by the preprocessing sequence. We compared resampling before the train/test split with resampling after the split, using three classifiers (DT, BaggingDT, and LightGBM), evaluated on three classifiers (BaggingDT, DT, and LightGBM), utilizing the CICIoT2023 dataset with 10-fold cross-validation repeated twice. The results show a significant increase in the scores of all three classifiers: accuracy is inflated by 9.09, 8.09, and 7.40 percentage points for BaggingDT, DT and LightGBM, respectively, and by 0.1518, 0.1707 and 0.1367 percentage points for macro-F1. Macro-F1 shows a greater increase than accuracy because SMOTE-ENN generates examples predominantly for the minority class, and therefore, the leakage disproportionately benefits the rare attack categories. Because macro-F1 assigns each class equal weight, this effect is reflected more strongly there than in accuracy. The differences are statistically considerable for all models ($p=2.0\times 10^{-6}$, paired Wilcoxon signed-rank test over folds). Given the above results, it was decided to apply resampling within the training folds of cross-validation. Figure 4 illustrates the effect that the preprocessing order has on performance with the CICIoT2023 dataset. The result of applying resampling before the split is presented in red bars, and the results for resampling within each training fold are shown by green bars. The dotted line marks an accuracy of 0.95 for reference.

### 5.3. Single Learners and Ensemble Performance

The following are the results of tests to establish whether it is better to add multiple classifiers to a soft-voting ensemble or employ a single tabular learner classifier.

Using the assessment process outlined in Section 4.3, Table 6, Table 7 and Table 8 present the performance of all models with the three datasets. Per-class one-vs-rest accuracy might be deceptive in unbalanced multiclass setups; for example, a per-class precision of just 0.61 may coexist with a one-vs-rest accuracy exceeding 0.98. For this reason, macro-averaged metrics are used instead. Table 6 presents the classification performance of each classifier and ensemble model on the CICIoT2023 dataset, using stratified 10-fold cross-validation repeated twice (20 fold-estimates per model). The classifier LightGBM achieved the highest accuracy (91.43%) and F1-score (85.06%), performing better than the standalone models. The proposed SoftVote-2 (BagDT + LightGBM) ensemble increased performance even further, achieving the highest accuracy (91.87%), precision (83.30%), recall (88.60%), F1-score (85.63%), and AUC (98.99%). This is a significant improvement over LightGBM for this dataset (McNemar’s test, *p* = 0.019). It is worth noting that SoftVote-2 produced the lowest benign false-positive rate (9.53%), i.e., it had the lowest number of false alarms of all the models assessed.

A comparison of the performance of individual ML models and ensemble methods on the TON_IoT dataset is presented in Table 7, using stratified 10-fold cross-validation repeated twice, giving 20-fold estimates per model. From the standalone classifiers, LightGBM was the most accurate (97.36%) and had the highest F1-score (94.11%); XGBoost scored the highest AUC (99.76%), and Random Forest had the lowest benign false-positive rate (0.77%). The proposed SoftVote-2 (BagDT + LightGBM) ensemble was competitive on all the evaluation metrics: accuracy 97.32%, precision 92.82%, recall 96.41%, F1-score 94.03%, and AUC 99.65%. It was only 0.04 percentage points less accurate than LightGBM, and McNemar’s test on the held-out partition revealed no statistically significant differences between these two on this particular dataset (*p* = 0.814, Section 5.6).

Table 8 presents a summary of the performance of the evaluated classifiers on the Edge-IIoTset dataset, using stratified 10-fold cross-validation repeated twice (20 fold-estimates per model). CatBoost was the highest-achieving individual classifier in terms of accuracy (88.34%), precision (85.73%), and recall (87.30%), while LightGBM scored the highest F1-score (84.82%), and XGBoost the highest AUC (99.18%). The proposed SoftVote-2 (BagDT + LightGBM) ensemble was competitive, with an accuracy of 88.10%, precision of 85.07%, F1-score of 84.47%, and AUC of 98.84%. Statistically, McNemar’s test showed no significant differences between the proposed ensemble and the strongest single classifier on this dataset (*p* = 0.709, see Section 5.7). All tree-based models, including SoftVote-2, achieved a zero false-positive rate on benign traffic in the held-out evaluation.

**Figure 5:**
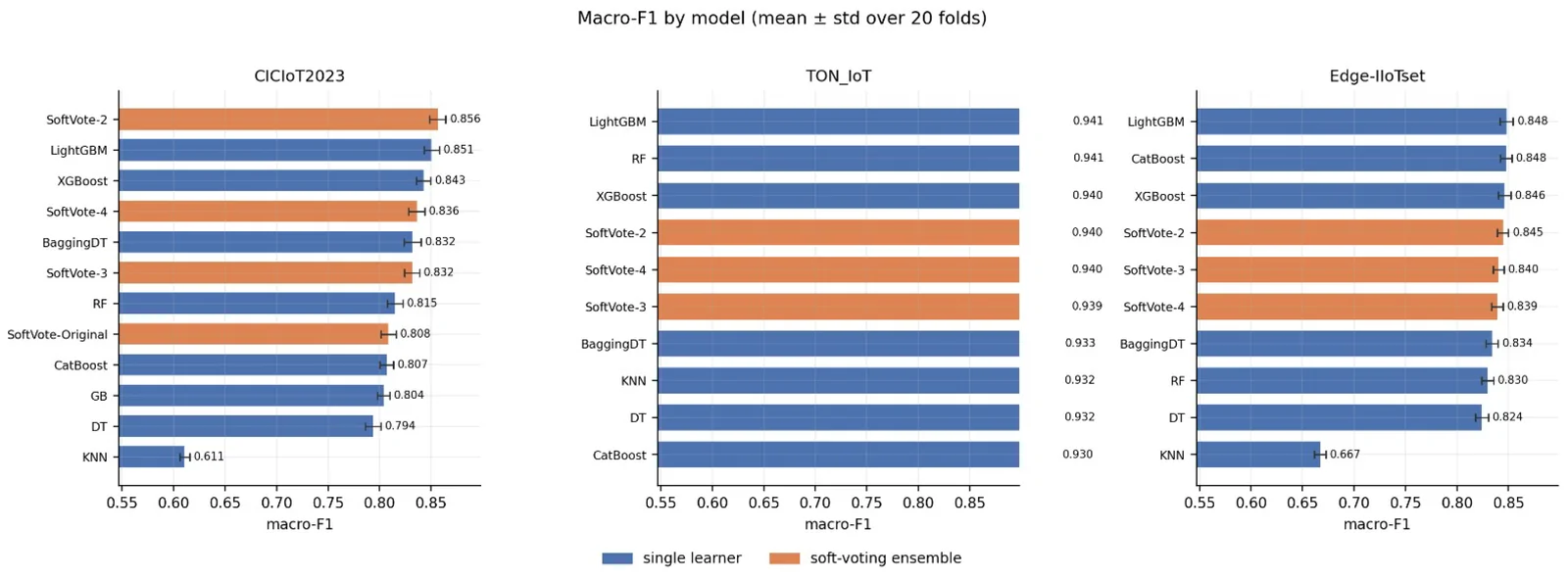
Macro-F1 of every model on each dataset. The error bars show the standard deviation across folds, with standalone learners colored blue and soft-voting ensembles colored orange.

The Macro-F1 of every model on each dataset is illustrated in Figure 5. The error bars show the standard deviation across folds, with standalone learners colored blue and soft-voting ensembles colored orange.

### 5.4. Ablation Studies

A number of ablation studies were carried out to assess the value of each part of the proposed approach. Recent ablation-driven analysis by Wicaksono et al. [45] presented that statistical traffic representations can lead to very strong performance in IoT DDoS detection. However, increasing deep learning complexity does not achieve proportional representational gains in IoT intrusion detection systems. These findings motivate a related investigation in the present study. We study whether combining multiple classifiers provides more enhancement over a single tabular learner such as LightGBM, instead of assuming that increasing model complexity will essentially lead to better performance. The previous section showed that the ensemble did not always perform better than LightGBM, so this section investigates whether its predictive value can be increased by adding more ensemble members. The analysis focuses on SoftVote-2, SoftVote-3, and SoftVote-4, which progressively increase ensemble size by adding different base learners. In order to assess the impact of ensemble size, we evaluated SoftVote configurations containing two, three, and four base learners under identical experimental conditions. The two-member SoftVote-2 configuration often offered the best performance, obtaining the greatest Macro-F1 on CICIoT2023 and Edge-IIoTset, as shown in Table 9, while every configuration performed similarly on TON_IoT. The combined findings show that an ensemble’s usefulness depends on whether its predictive gain is significant enough to outweigh its extra expense. SoftVote-2 raises macro-F1 for LightGBM from 0.8506 to 0.8563 on CICIoT2023. This is a 0.0057 absolute improvement. In light of this relatively small gain, the higher processing cost of SoftVote-2 should be taken into account when making comparisons. The scenario is different for Edge-IIoTset and TON_IoT. The assessed ensemble configurations are already matched or surpassed by LightGBM; therefore, greater ensemble complexity does not yield a comparable increase in prediction. The findings, therefore, show that rather than relying solely on predictive performance, ensemble selection should be based on a performance–complexity trade-off. Only when the extra predictive data from a more complicated model offer a sufficiently significant advantage for the intended use is it justified. These findings imply that a well-chosen subset of complementary learners may be more successful than a larger ensemble and that adding more classifiers does not always increase ensemble performance.

Table 10 compares the best ensemble with the best single learner. The ensemble achieves a higher Macro-F1 on CICIoT2023, whereas LightGBM performs slightly better on TON_IoT and Edge-IIoTset. Table 10 provides an overview of the macro-F1 findings for the three ensemble configurations and the best single learner across the three datasets. For every dataset, the setup that performs the best is displayed in bold.

**Figure 6:**
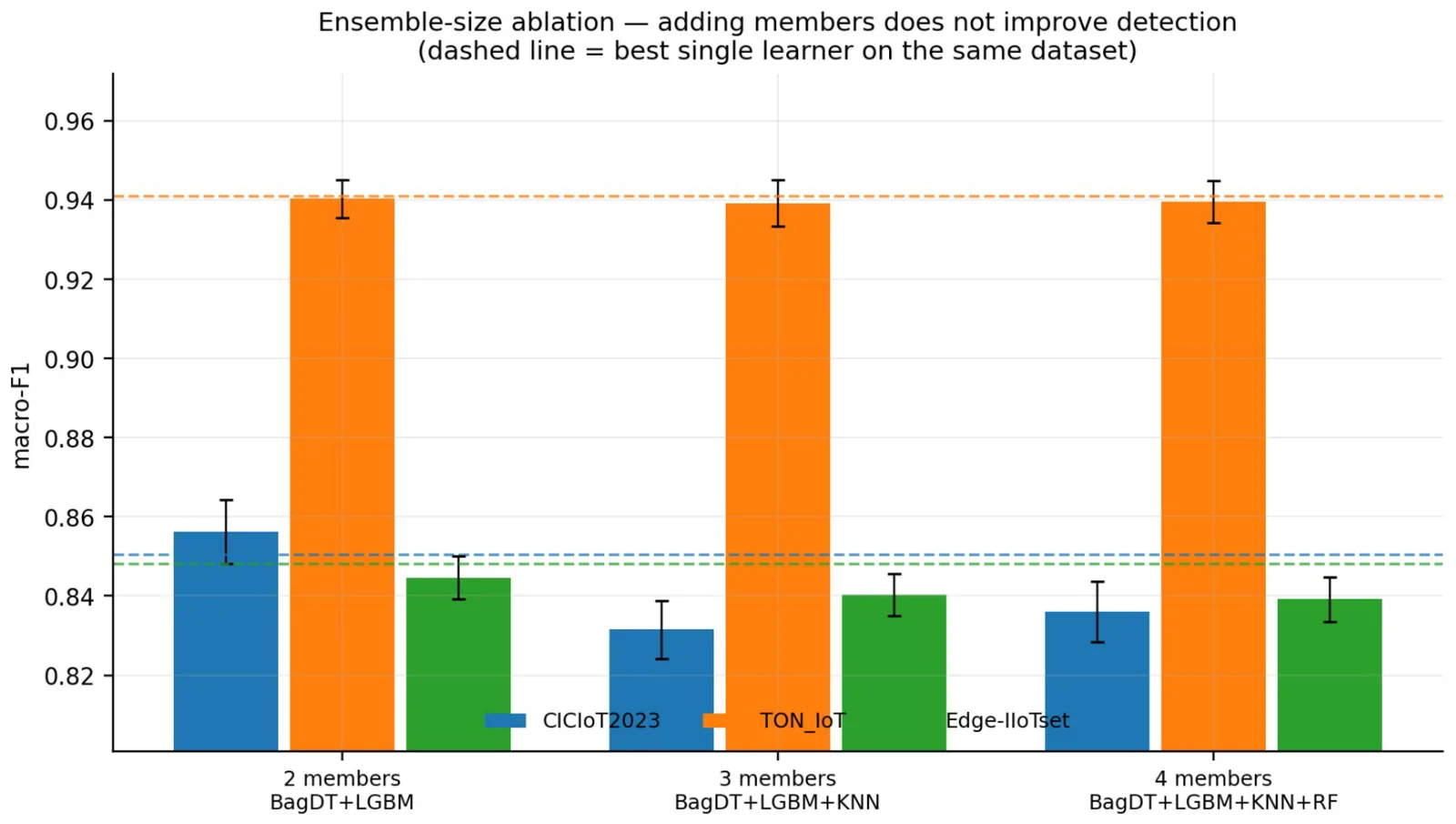
Ensemble-size ablation. Bars show macro-F1 for ensembles of two, three and four members on each dataset; the dashed horizontal line marks the best single learner on the corresponding dataset. Note that the vertical axis is truncated in order to make differences of a few thousandths visible.

Table 9 and Figure 6 point to two consistent findings across all three datasets. First, adding more members does not improve performance; the two-member ensemble matches or exceeds the three- and four-member versions in every case, meaning that extra members add computational overhead without any gain in detection. Including kNN as a third member is particularly harmful on CICIoT2023. Second, the ensemble does not consistently beat its strongest individual member; where it does perform better, the margin is comparable to the fold-to-fold standard deviation. The corresponding significance tests are reported in Section 5.7. Soft voting is commonly used in intrusion-detection research on the assumption that combining learners helps, but this benefit depends on the members making weakly correlated errors. Since a modern gradient-boosting implementation is already a strong single learner, this is often not the case, meaning that for resource-constrained IoT deployments, a single well-chosen model may perform better in terms of both accuracy and cost (see Table 11).

### 5.5. Computational Cost and Deployment Considerations

To evaluate how practical the proposed ensemble framework would be in resource-constrained IoT and edge settings, we also examined its computational cost and deployment requirements. As well as predictive performance, factors like training time, inference latency, memory consumption, and model size all affect the feasibility of deploying ML models on real-world systems. Table 12 summarizes the computational characteristics of the individual learners and ensemble configurations on the CICIoT2023 dataset. Since all measurements were taken under the same hardware and experimental conditions, the computational overhead of each model can be compared consistently. The findings clearly illustrate the trade-off between classification performance and deployment efficiency, especially when choosing an ensemble configuration for edge or resource-constrained applications.

Table 12 lists two latency measures for each model, reflecting several deployment scenarios. Batch inference latency captures the amortized cost of processing multiple samples and is, therefore, most relevant to offline or batch-oriented analysis, while single-sample latency reflects the time needed to classify an individual sample. This matters most for inline or real-time intrusion detection, where each network flow must be classified as it arrives. The table also distinguishes retained memory from training-time memory consumption: retained memory refers to what remains allocated after model fitting, making it a more direct indicator of the memory a deployed gateway or edge device would need to keep the model resident during inference. Together, these measurements offer a more deployment-oriented view of the computational and resource demands of the evaluated models. Table 12 presents these figures for each model.

**Figure 7:**
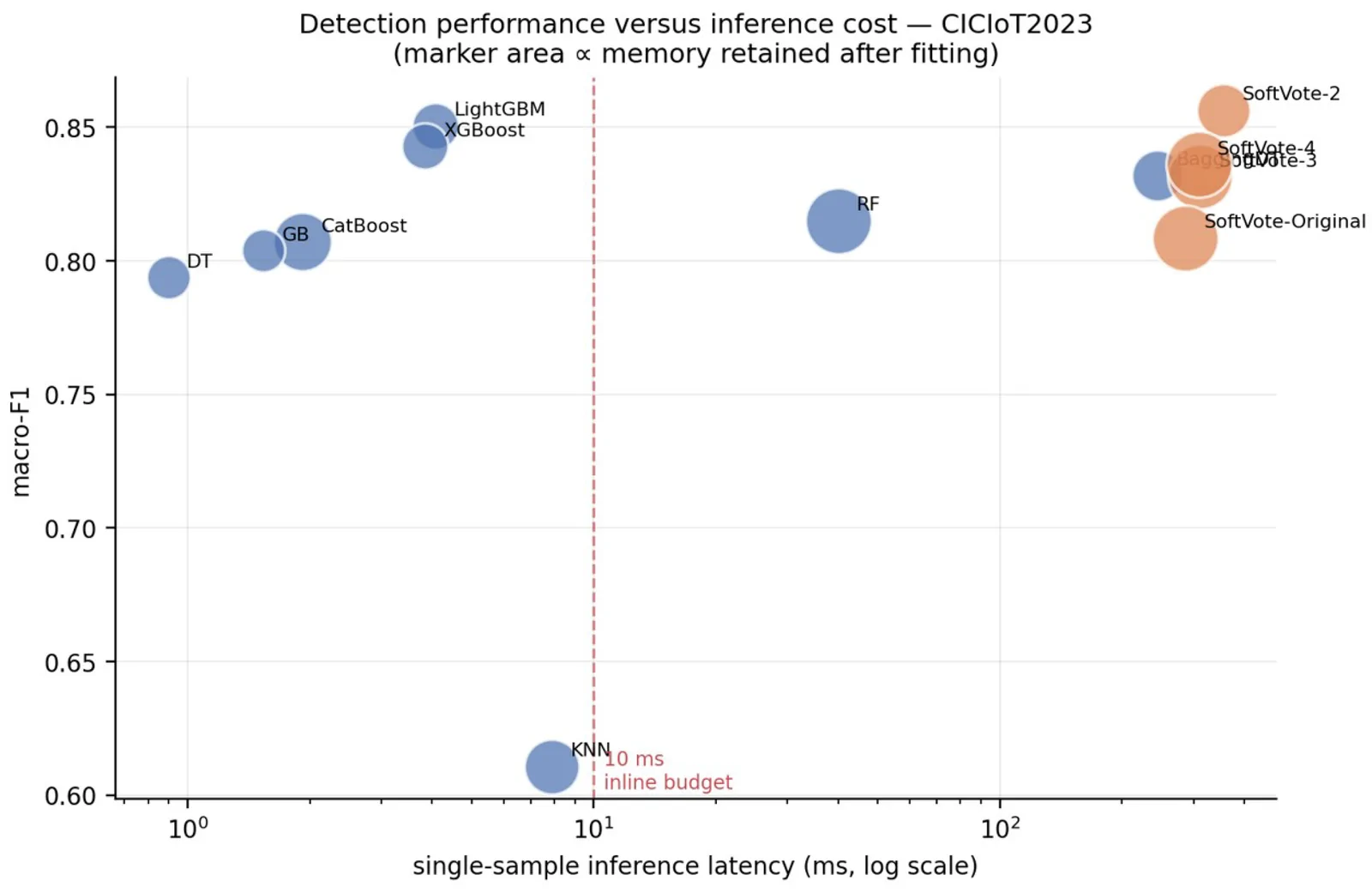
Detection performance against inference cost on CICIoT2023. The horizontal axis is single-sample latency on a logarithmic scale, and marker area is proportional to the memory retained after fitting. The dashed vertical line marks a 10 ms target for inline deployment.

This trade-off is shown in Figure 7, where the boosted single learners easily satisfy the 10 ms single-sample budget while voting ensembles and BaggingDT lie to the right of it. The cost profile of kNN is an additional deployment limitation. In contrast to tree and boosting models, which discard their training data once fitted, this lazy learner keeps the full training set in order to respond to questions, and its per-query cost increases with the size of that set. This is a strong case for removing kNN from a deployed configuration when combined with the ablation result showing that it does not increase ensemble accuracy. If a distance-based member is still needed, the reduced feature set of Section 5.6 or approximate nearest-neighbor indexing would lower the expense.

### 5.6. Model Explainability with LIME

Model explainability is essential for understanding how ML models arrive at their predictions, and can be assessed at either a global or local level. LIME is focused specifically on local explainability: it makes complex models more interpretable by fitting a simple model around one specific prediction, which is particularly useful for producing quick, intuitive explanations for individual cases. SHAP (Shapley Additive Explanations), by contrast, offers both global and local insight. At the global level, it assigns Shapley values to quantify each feature’s contribution across the model as a whole; at the local level, it explains individual predictions by calculating each feature’s contribution for that specific instance, giving a fuller and more balanced picture of model behavior.

In this study, LIME was applied to assess the model’s interpretability across different attack types. Examining the model’s explanations for various instances gave insight into how it processes and detects each specific attack type.

These instance-level explanations are illustrative on their own, but they cannot establish whether the explanations hold up under LIME’s stochastic sampling, whether they are faithful to the model they are meant to explain, or whether they agree with an independent attribution method. We, therefore, measure all three properties directly, and additionally test whether the explanations are operationally useful. To quantify these properties, we performed the following evaluations:

- Stability: LIME is run ten times on each of 200 stratified test instances, with the mean pairwise Jaccard overlap of the top-seven feature sets reported per class and overall.
- Fidelity: Two complementary measures are used: (i) the local surrogate model’s $R^{2}$, capturing how well the surrogate approximates the original model locally, and (ii) a deletion test in which the top-*k* features identified by LIME are replaced with their median values, with the resulting drop in predicted probability compared against the drop from masking *k* randomly selected features instead.
- Cross-method agreement: LIME’s aggregated feature attributions are compared against global SHAP values computed exactly for the tree-based ensemble member, with agreement quantified via top-*k* Jaccard overlap and Kendall’s τ.
- Practical usefulness: The model is retrained on only the fifteen highest-attribution features, and detection performance is evaluated alongside the resulting reductions in inference latency and model size.

Ready for the next section.

The three metrics for the suggested ensemble are shown in Table 13 on the three datasets. Stability is poor on TON_IoT and Edge-IIoTset (0.21 and 0.36) and moderate on CICIoT2023 (top-7 Jaccard of 0.53): A single LIME output should not be regarded as the explanation of a prediction, as successive explanations of the same event often contradict each other. On the other hand, fidelity is consistently high: the explanations do refer to aspects that the model really depends on since masking the features that LIME finds most significant decreases the predicted probability 4.8 to 7.0 times more than masking randomly selected features. Given the disparate attribution processes of the two approaches, the partial agreement with SHAP (Jaccard 0.11–0.43) is expected.

The models were retrained using the fifteen highest-attribution characteristics found by the XAI study in order to assess if the explanations can support both interpretability and model efficiency. Retraining on these features reduces inference latency and model size while maintaining between 98.4% and 99.4% of macro-F1, as seen in Table 14. The impact is particularly noticeable on TON_IoT, where latency is reduced by 38.0% and model size is reduced from 147.7 MB to 40.3 MB, while only 15 of 124 features (12.1%) retain 99.4% of macro-F1. Therefore, explainability directly affects the detector’s effectiveness.

Table 15 shows the top 10 global features for each dataset according to the mean absolute SHAP value. The characteristics found align with the mechanisms of the aforementioned assaults. The rankings on CICIoT2023 and TON_IoT are dominated by flow-timing and volume descriptors, inter-arrival time, header length, packet counts, and aggregate size. This is expected because timing regularity and volume, rather than packet content, are the main ways that flooding attacks differentiate themselves from benign traffic. The transport-specific variations of the same attack family are distinguished by protocol indicator variables. A sanity check on the explanations is provided by the correspondence between the attributions and the known signatures of the attack classes. Even if a model’s accuracy was high, it would be questionable if it depended on attributes that had no conceivable connection to the attack mechanism.

#### Comparison of Explanation Consistency Between Models

A model is more trustworthy to act on when its local, instance-specific reasoning echoes the same features driving its overall behavior; that consistency is what gives an analyst confidence in an individual alert. With that in mind, we benchmark the proposed ensemble (SoftVote-2: BagDT + LightGBM) against LightGBM alone, the best-performing single model. For each of the two models, SHAP is used to identify the twenty globally most influential features; one representative instance is then drawn per class from the held-out data, LIME explanations are generated for each, and we calculate what fraction of the ten features LIME ranks most influential locally overlap with that model’s top twenty global features.

**Figure 8:**
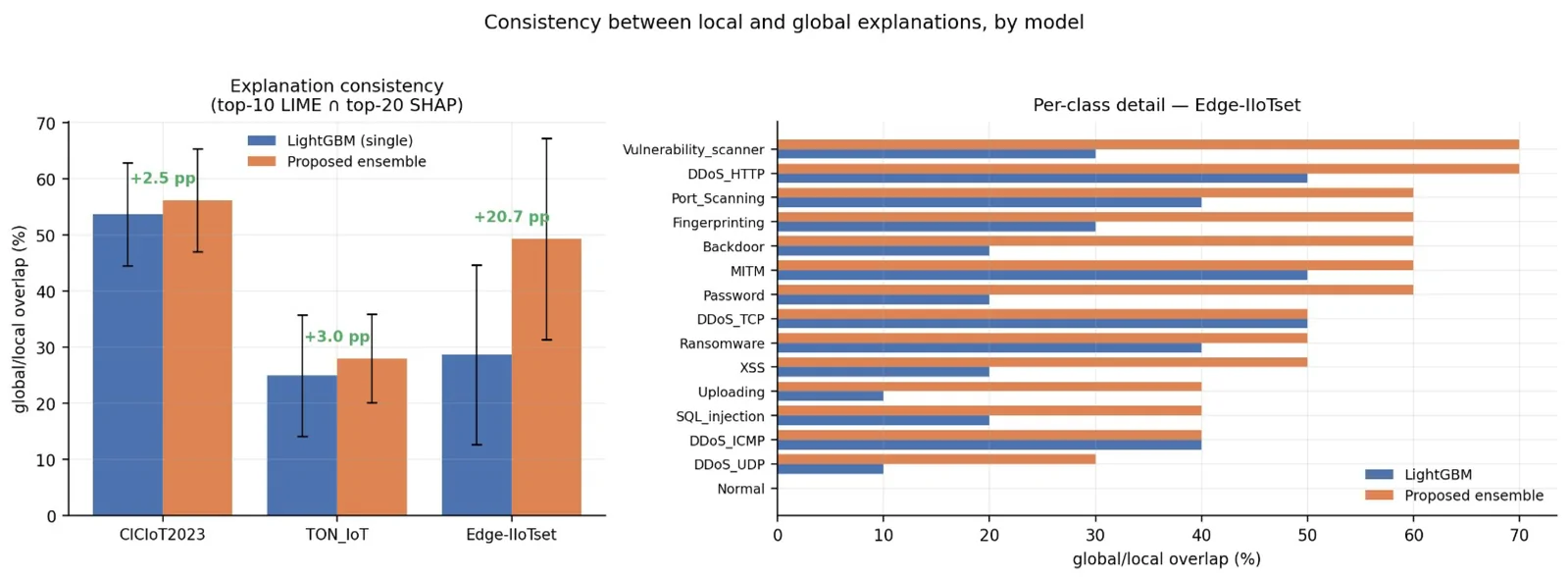
Consistency between local and global explanations for the proposed ensemble (SoftVote-2: BagDT + LightGBM) and LightGBM. (**Left**) mean proportion of each instance’s ten most influential LIME features that also appear among the model’s twenty most important features globally, with error bars showing the standard deviation across instances. (**Right**) per-class detail for the dataset with the largest difference between the two models.

To evaluate this consistency more broadly, global SHAP rankings (top-20) are compared against local LIME rankings (top-10) across the framework as a whole (See Figure 8).

As reported in Table 16, the proposed ensemble performs better on all three datasets, with a higher global/local overlap than the LightGBM baseline. The overlap reaches 56.2±9.2% on CICIoT2023, 28.0±7.9% on TON_IoT, and 49.3±17.9% on Edge-IIoTset, compared with 53.8±9.2%, 25.0±10.8%, and 28.7±16.0%, respectively, for LightGBM. In each dataset, the suggested ensemble also achieves higher surrogate $R^{2}$ values, suggesting that the local explanations offer a more accurate approximation of the underlying model behavior. These findings imply that the suggested framework generates explanations that are more compatible with the global feature-attribution patterns in addition to being locally faithful.

This comparison is subject to one methodological qualification. As a result, both models are evaluated against the same global feature reference, and the twenty-feature sets overlap. The ensemble’s global SHAP attributions are calculated on its gradient-boosting component, which is precise and affordable with a tree architecture. Therefore, the comparison should not be seen as two independently generated rankings converging, but rather as a measure of how closely each model’s local explanations match a common global reference. This condition would be eliminated at a significantly higher computational cost by obtaining a fully model-agnostic attribution for the ensemble using a kernel-based estimate over all members. Together with the actual class and the model’s classification choice, Table 17 summarizes the findings for the chosen examples and shows the characteristics that had the biggest and least impact on the decision-making process. The most important characteristics differ depending on the kind of assault. Protocol Type and Magnitude are crucial for Mirai, IAT and DNS are crucial for Web assaults, and ICMP and SSH are crucial for Recon. These results show that rather than depending on a single global pattern, the model relies its predictions on characteristics that are typical of each assault type.

### 5.7. Statistical Significance

To control the family-wise error rate under multiple comparisons, we employ a Friedman test with Nemenyi post-hoc analysis across all models concurrently, a paired Wilcoxon signed-rank test across folds for pairwise comparisons, and McNemar’s test on the unaltered test partition. Because it assesses predictions on data that were not used in any cross-validation fold, the latter offers the deployment-relevant comparison. At α=0.05, statistical significance is evaluated, and precise *p*-values are shown. The findings of the corresponding statistical significance testing are shown in Table 18.

## 6. Discussion

As noted earlier, we proposed an AI-based model for intrusion attack detection. Drawing on the results presented in the previous section, we applied multi-class classification across 8 classes: 7 abnormal classes plus one normal class. Bagging Decision Trees, kNN, and Gradient Boosting Classifier were used as baselines against which to compare our proposed work. Bagging Decision Trees were chosen for their ability to reduce the variance of Decision Trees, which are otherwise prone to overfitting [46,47]. kNN was selected for its simplicity and widespread use in predictive analysis [47]. Gradient Boosting Classifier serves as a further point of comparison, given its strength as a stand-alone predictor and its competitiveness in machine learning more broadly, particularly when training data, training time, or expertise for parameter tuning are limited [48].

While some research uses statistical, temporal, or hybrid representations to improve the quantity or kind of information accessible to the classifier, others use deep, adaptive, or more complicated structures to capture complex traffic patterns. This prompted us to investigate a different type of complexity in the current work by merging several powerful tabular classifiers and assessing if the ensuing prediction benefits are significant enough to warrant the extra costs associated with computing, deployment, and interpretability. Therefore, the research focuses on determining the circumstances in which multiple classifiers give complementary prediction value and when a powerful single learner, like LightGBM, is already adequate, rather than assuming that greater complexity inevitably leads to better detection. As shown previously in Table 6 for the CICIoT2023 dataset, SoftVote-2 increased the accuracy from 91.43% to 91.87%, recall from 87.85% to 88.60%, and F1-score from 85.06% to 85.63%, compared with LightGBM (statistically significant, McNemar’s test, *p* = 0.019) (see Section 5.7). This suggests that the combination of Bagging Decision Trees with LightGBM allows the ensemble to exploit complementary decision boundaries, which leads to improved generalization compared with either model on its own. Furthermore, the results indicate that the addition of other base learners such as Random Forest and KNN was not beneficial, as SoftVote-2 consistently outperformed the other ensemble variants (SoftVote-Original, SoftVote-3, and SoftVote-4). This confirms that the best approach is to employ a carefully selected ensemble composed of complementary classifiers, rather than larger ensembles containing weaker or redundant models.

Based on the results in Table 7 for TON_IoT, SoftVote-2 consistently outperformed SoftVote-3 and SoftVote-4 in F1-score relative to the other ensemble models, while maintaining a low false positive rate (0.0099). It also combines the strengths of Bagging Decision Trees and LightGBM, yielding robust and stable predictions that do not depend on a single classifier. This points to the ensemble striking an effective balance between predictive performance and generalization, making it a dependable choice for this classification task.

Turning to Table 8 for Edge-IIoTset, SoftVote-2’s comparison against its constituent members shows that combining Bagging Decision Trees with LightGBM strengthens the model’s ability to correctly identify attack instances, reflected in its stronger recall and overall accuracy. CatBoost edges out the ensemble slightly on accuracy, precision, recall, and F1-score, and XGBoost achieves the highest AUC, but these differences are small and not statistically significant (McNemar’s test, *p* = 0.709, Section 5.7). SoftVote-2, therefore, remains statistically comparable to the strongest single learners on this dataset, while producing the substantially more consistent explanations reported in Section Comparison of Explanation Consistency Between Models. Soft voting did not consistently outperform its strongest single member on any of the three datasets, and expanding the ensemble did not give any additional benefit. Therefore, this work’s contribution does not constitute an assertion of ensemble supremacy. Instead, the evaluation protocol allows for the confident statement of three conclusions: that an easily overlooked ordering decision in the preprocessing pipeline materially inflates reported performance; that the benefit of soft voting for this task is conditional and, for the member sets examined here, largely absent once a modern gradient-boosting learner is included and that explanation quality can be measured rather than merely illustrated, with implications for how much weight an analyst should place on a single explanation. To understand the factors influencing the classification process, LIME was applied to provide deeper insight into the model’s decision-making. As discussed in Section 5.6, Table 13, Table 14, Table 15, Table 16 and Table 17 offer quantitative evidence that LIME explanations are reasonably stable, informative, and practically useful, while also showing that explanation consistency varies by dataset. Table 13 shows Top-7 Jaccard stability ranging from 0.214±0.023 on TON_IoT to 0.528±0.078 on CICIoT2023, with corresponding surrogate $R^{2}$ values between 0.171 and 0.208; notably, feature-selection agreement is consistently higher than what would be expected from random rankings. This is reinforced by the comparison with SHAP in Table 16, where the proposed model shows greater LIME-SHAP overlap and higher surrogate $R^{2}$ than LightGBM across all three datasets, though the degree of agreement differs by dataset. Table 15 indicates that globally important features are dataset-specific: traffic timing and packet statistics dominate on CICIoT2023, source/destination traffic characteristics dominate on TON_IoT, and protocol- and transport-layer features stand out on Edge-IIoTset. This alignment between global SHAP structure and local LIME explanations is further supported by the practical results in Table 14, where narrowing the input to the 15 highest-attribution features preserves 98.4–99.4% of the original macro-F1 while cutting latency by up to 38.0% and substantially reducing model size. The instance-level examples in Table 17 illustrate further how LIME pinpoints influential features behind individual predictions.

For example, features such as ICMP, SSH, and fin_flag_number had a major impact on the predictions for Recon attacks, while for Web attacks, the most influential features were IAT, DNS, and ICMP. In the case of Mirai, features such as Protocol Type and Magnitude played the most significant roles in prediction. However, arp, rst_flag_number, and ack_count had less impact across all three instances.

Applying LIME increases the trust level in the model’s decisions. Overall, the results show that LIME offers explanations that are both useful and partially consistent with SHAP, with value extending beyond interpretation into feature reduction and deployment optimization—while the variation observed in stability and cross-method agreement underscores the need to assess explanation reliability quantitatively rather than relying on isolated examples. Table 18 shows that the performance gaps between the ensemble configurations and LightGBM are mostly statistically significant, though what matters more is their direction and practical size. On CICIoT2023, every SoftVote configuration differs significantly from LightGBM under the Wilcoxon test: SoftVote-2 has a small positive mean difference of +0.00569, while SoftVote-3 and SoftVote-4 perform significantly worse. On TON_IoT, all three ensembles differ significantly from LightGBM, but in each case the mean difference is negative, meaning LightGBM performs better; notably, the McNemar test comparing SoftVote-2 to LightGBM is not significant (p=0.814). Edge-IIoTset shows a similar picture: SoftVote-2 is not significantly different from LightGBM (p=0.0825), whereas SoftVote-3 and SoftVote-4 are significantly worse. Friedman tests likewise confirm significant differences among the models on all three datasets (p<0.001). Taken together, these results show that statistical significance does not necessarily translate into a practically meaningful ensemble advantage: only CICIoT2023 yields a statistically significant gain for SoftVote-2, whereas on the other two datasets LightGBM alone matches or significantly outperforms the ensembles.

## 7. Conclusions

AI models used in IDS are often not adequately interpreted, which motivates the model proposed in this paper: an AI-based system for detecting and classifying cyberattacks in IoT environments. The model takes the form of a hybrid ensemble, combining Bagging Decision Trees, k-Nearest Neighbors, and Gradient Boosting via soft voting. It was assessed using a leakage-free protocol across three IoT datasets (CICIoT2023, TON_IoT, and Edge-IIoTset) and benchmarked against Random Forest, LightGBM, XGBoost, and CatBoost under identical conditions.

On CICIoT2023, the ensemble achieves an accuracy of 0.9187 ± 0.0044. As shown in Section 5.1, applying resampling before the train/test split instead would inflate accuracy by roughly eight percentage points and macro-F1 by fourteen to seventeen points—which is the rationale for using the leakage-free protocol throughout.

Given the imbalanced nature of the dataset, SMOTE-ENN was used to address class imbalance and improve the reliability of predictions; its contribution, alongside that of the individual ensemble members, is quantified in the ablation study in Section 5.3. LIME was also applied for explainability, offering transparent insight into the features driving attack classification decisions and supporting greater trust in the system.

Three findings stand out. First, the order in which preprocessing steps are applied has a substantial effect on reported performance, large enough to alter conclusions. Second, soft voting did not consistently outperform its best individual member, and increasing ensemble size brought no additional benefit; for this task, a single well-tuned gradient-boosting model performs competitively with the ensembles tested at lower cost. Third, evaluating explanations as measurable properties rather than treating them as illustrative changes what can legitimately be claimed about them. LIME explanations also showed measurable stability, non-random agreement between features, and partial alignment with SHAP, while proving useful for feature reduction. Statistical testing further indicated that the benefits of ensembling are dataset-dependent: only SoftVote-2 achieved a significant improvement over LightGBM on CICIoT2023, whereas LightGBM matched or outperformed the ensembles on TON_IoT and Edge-IIoTset. This suggests that added ensemble complexity should be adopted selectively, based on demonstrated marginal gains rather than assumed to be beneficial.

Overall, the approach shows promise for strengthening IoT cybersecurity and provides a basis for future work involving additional explainability techniques and larger datasets.

These findings come with several caveats. The evaluation was conducted offline using flow-level features, with latency measured on a single CPU configuration rather than across varied hardware. All three datasets were subsampled to 66,000 instances, and the CICIoT2023 subset is heavily skewed toward benign traffic, which may limit how representative it is of real-world conditions. Additionally, while LIME and SHAP indicate which features influence predictions, they do not establish causal explanations for attack behavior. The ensemble’s performance gain over the strongest individual model is also modest, so it remains unclear whether this advantage would hold across different model combinations or attack taxonomies. Concept drift and adversarial robustness fell outside the scope of this evaluation.

Building on this, future work will test the framework’s performance under concept drift and adversarial conditions, explore calibrated or weighted approaches to ensemble voting, and assess latency and resource use on hardware representative of real gateway deployments. 

## Figures and Tables

**Table 1:** Comparison of recent IoT intrusion detection studies and the proposed approach.

Ref.	Approach	Accuracy	Macro-F1	Protocol	Ensemble	XAI Method
[11]	RF, AdaBoost, XGB, LGBM, CB	98.54% (XGB)	NR	Two public datasets; split not fully specified	Bagging	Rule Induction
[12]	CNN + LSTM	90%	NR	DDoS detection	Attention Fusion	SHAP
[13]	CatBoost	99.96%	NR	Attack subtypes; split not specified	No	SHAP
[15]	RF with LLM agent	99.97%	NR	Split not fully specified	No	SHAP, LIME
[16]	CNN + BiLSTM	85.56%	NR	Split not fully specified	No	SHAP, LIME
[20]	XGBoost, LightGBM, CatBoost	0.9959	**0.8903**	8-class CICIoT2023; 5-fold CV	No	Feature importance
[21]	Balanced RF, LightGBM, CatBoost, Extra Trees, XGBoost	0.9916	**0.7379**	33-class CICIoT2023; hybrid feature selection	Yes	SHAP + LIME
[22]	LightGBM, Random Forest, XGBoost	0.9903	**0.9694**	TON_IoT multiclass; 80/20 stratified split	No	Feature importance
**This study**	Bagging DT, KNN, GB, RF, LGBM, XGB, CB	CICIoT2023; TON_IoT; Edge-IIoTset (Table 7, Table 8 and Table 9)	**0.8563/0.9403/0.8447**	8-class; benign-heavy subsample; 10-fold CV × 2; leakage-free; three datasets	Soft Voting	LIME + SHAP, quantified

*Notes:* NR = not reported. Reported F1 values reproduce the metric reported in the corresponding study and are not necessarily macro-F1. Therefore, these values should not be interpreted as a direct performance ranking across studies because the datasets, classification tasks, preprocessing procedures, and evaluation protocols differ. *Abbreviations:* RF, Random Forest; DT, Decision Tree; KNN, K-Nearest Neighbors; GB, Gradient Boosting; XGB, XGBoost; LGBM, Light Gradient Boosting Machine; CB, CatBoost; CNN, Convolutional Neural Network; LSTM, Long Short-Term Memory; BiLSTM, Bidirectional Long Short-Term Memory; SHAP, SHapley Additive exPlanations; LIME, Local Interpretable Model-Agnostic Explanations.

**Table 2:** Subcategories of each attack type in the CICIoT2023 dataset [10].

DDoS	Brute Force	Spoofing	DoS	Recon	Web-Based	Mirai
ACK Fragmentation	Dictionary Brute Force	ARP Spoofing	TCP Flood	Ping Sweep	SQL Injection	GREIP Flood
UDP Flood		DNS Spoofing	HTTP Flood	OS Scan	Command Injection	GREETH Flood
Slowloris			SYN Flood	Vulnerability Scan	Backdoor Malware	UDPPlain
ICMP Flood			UDP Flood	Port Scan	Uploading Attack	
RSTFIN Flood				Host Discovery	XSS	
PSHACK Flood					Browser Hijacking	
HTTP Flood						
UDP Fragmentation						
TCP Flood						
SYN Flood						
SynonymousIP Flood						

**Table 3:** Summarises the key characteristics of the datasets [10,41,42,43] used in this study.

Dataset	Rows	Features	Classes	Majority Class
CICIoT2023 (8-class)	66,000	46	8	Benign (50.0%)
TON_IoT	66,000	124	10	Normal (23.7%)
Edge-IIoTset	66,000	59	15	Normal (15.4%)

**Table 4:** Model configurations evaluated under a consistent preprocessing pipeline, cross-validation strategy, and random seed.

Group	Models and Settings
Original base learners	Decision Tree; Bagging Decision Tree (100 estimators); kNN (k=7); Gradient Boosting (default settings)
Added baselines	Random Forest (200 trees); LightGBM (300 iterations, learning rate 0.1); XGBoost (300 iterations, learning rate 0.1); CatBoost (300 iterations, learning rate 0.1)
Soft-voting ensembles	Original: Bagging DT + kNN + GB; E2: Bagging DT + LightGBM; E3: Bagging DT + LightGBM + kNN; E4: Bagging DT + LightGBM + kNN + RF

**Table 5:** Effect of preprocessing order on CICIoT2023 under 10-fold cross-validation with two repeats.

Model	Resample Before Split	Resample Within Folds	Δ Accuracy	Δ Macro-F1	*p*
DT	0.9658±0.0015	0.8749±0.0056	+0.0909	+0.1707	$2.0\times 10^{-6}$
BaggingDT	0.9845±0.0007	0.9035±0.0054	+0.0809	+0.1518	$2.0\times 10^{-6}$
LightGBM	0.9884±0.0009	0.9144±0.0046	+0.0740	+0.1367	$2.0\times 10^{-6}$

**Table 6:** CICIoT2023: mean ± standard deviation over 20 folds.

Model	Accuracy	Precision (Macro)	Recall (Macro)	F1 (Macro)	AUC (Macro)	FPR (Benign)
DT	0.8749±0.0056	0.7605±0.0069	0.8521±0.0082	0.7937±0.0074	0.9172±0.0043	0.1522±0.0089
BaggingDT	0.9035±0.0054	0.8009±0.0085	0.8774±0.0081	0.8319±0.0083	0.9852±0.0020	0.1173±0.0086
KNN	0.6787±0.0048	0.5955±0.0033	0.6989±0.0092	0.6107±0.0047	0.8664±0.0059	0.4190±0.0081
GB	0.8788±0.0047	0.7682±0.0055	0.8821±0.0082	0.8038±0.0061	0.9871±0.0012	0.1654±0.0077
RF	0.8816±0.0043	0.7870±0.0068	0.8565±0.0100	0.8151±0.0076	0.9821±0.0019	0.1499±0.0067
LightGBM	0.9143±0.0044	0.8288±0.0076	0.8785±0.0082	0.8506±0.0073	0.9893±0.0013	0.0984±0.0071
XGBoost	0.9097±0.0041	0.8145±0.0062	0.8827±0.0085	0.8428±0.0068	0.9893±0.0011	0.1084±0.0070
CatBoost	0.8818±0.0056	0.7711±0.0060	0.8795±0.0094	0.8070±0.0065	0.9871±0.0012	0.1582±0.0103
SoftVote-Original (BagDT + KNN + GB)	0.8809±0.0047	0.7720±0.0064	0.8742±0.0095	0.8085±0.0074	0.9835±0.0013	0.1604±0.0075
SoftVote-2 (BagDT + LGBM)	0.9187±0.0044	0.8330±0.0083	0.8860±0.0083	0.8563±0.0081	0.9899±0.0011	0.0953±0.0063
SoftVote-3 (BagDT + LGBM + KNN)	0.9015±0.0048	0.8012±0.0065	0.8755±0.0098	0.8315±0.0073	0.9838±0.0015	0.1222±0.0081
SoftVote-4 (BagDT + LGBM + KNN + RF)	0.9032±0.0048	0.8071±0.0070	0.8770±0.0096	0.8361±0.0077	0.9836±0.0017	0.1210±0.0072

**Table 7:** TON_IoT: mean ± standard deviation over 20 folds. Values marked with † are single-run estimates on the held-out partition rather than cross-validation means.

Model	Accuracy	Precision (Macro)	Recall (Macro)	F1 (Macro)	AUC (Macro)	FPR (Benign)
DT	0.9647±0.0031	0.9235±0.0069	0.9508±0.0085	0.9321±0.0062	0.9735±0.0044	0.0115 ^†^
BaggingDT	0.9657±0.0035	0.9238±0.0063	0.9528±0.0080	0.9329±0.0058	0.9851±0.0031	0.0109 ^†^
KNN	0.9694±0.0020	0.9199±0.0056	0.9590±0.0070	0.9322±0.0058	0.9813±0.0034	0.0131 ^†^
RF	0.9731±0.0021	0.9285±0.0056	0.9655±0.0067	0.9408±0.0058	0.9944±0.0012	0.0077 ^†^
LightGBM	0.9736±0.0019	0.9290±0.0051	0.9649±0.0068	0.9411±0.0052	0.9966±0.0012	0.0090 ^†^
XGBoost	0.9733±0.0019	0.9282±0.0053	0.9651±0.0065	0.9404±0.0053	0.9976±0.0007	0.0106 ^†^
CatBoost	0.9634±0.0023	0.9210±0.0056	0.9568±0.0057	0.9297±0.0060	0.9963±0.0009	0.0147 ^†^
SoftVote-2 (BagDT + LGBM)	0.9732±0.0019	0.9282±0.0048	0.9641±0.0060	0.9403±0.0047	0.9965±0.0013	0.0099 ^†^
SoftVote-3 (BagDT + LGBM + KNN)	0.9725±0.0020	0.9270±0.0054	0.9637±0.0077	0.9392±0.0058	0.9965±0.0013	0.0080 ^†^
SoftVote-4 (BagDT + LGBM + KNN + RF)	0.9727±0.0019	0.9273±0.0049	0.9643±0.0074	0.9396±0.0053	0.9955±0.0009	0.0080 ^†^

**Table 8:** Edge-IIoTset: mean ± standard deviation over 20-fold. Values marked with † are single-run estimates on the held-out partition rather than cross-validation means.

Model	Accuracy	Precision (Macro)	Recall (Macro)	F1 (Macro)	AUC (Macro)	FPR (Benign)
DT	0.8620±0.0051	0.8306±0.0047	0.8488±0.0065	0.8242±0.0062	0.9195±0.0034	0.0000 ^†^
BaggingDT	0.8714±0.0049	0.8403±0.0043	0.8584±0.0059	0.8340±0.0058	0.9580±0.0026	0.0000 ^†^
KNN	0.7096±0.0051	0.6690±0.0054	0.6871±0.0068	0.6670±0.0056	0.8763±0.0035	0.0015 ^†^
RF	0.8669±0.0048	0.8362±0.0046	0.8540±0.0059	0.8296±0.0059	0.9780±0.0038	0.0000 ^†^
LightGBM	0.8805±0.0045	0.8516±0.0051	0.8636±0.0097	0.8482±0.0063	0.9890±0.0015	0.0000 ^†^
XGBoost	0.8802±0.0039	0.8514±0.0043	0.8638±0.0076	0.8460±0.0061	0.9918±0.0008	0.0000 ^†^
CatBoost	0.8834±0.0046	0.8573±0.0043	0.8730±0.0058	0.8478±0.0056	0.9886±0.0010	0.0000 ^†^
SoftVote-2 (BagDT + LGBM)	0.8810±0.0043	0.8507±0.0041	0.8685±0.0058	0.8447±0.0054	0.9884±0.0016	0.0000 ^†^
SoftVote-3 (BagDT + LGBM + KNN)	0.8761±0.0046	0.8455±0.0045	0.8614±0.0076	0.8403±0.0052	0.9850±0.0016	0.0000 ^†^
SoftVote-4 (BagDT + LGBM + KNN + RF)	0.8757±0.0046	0.8448±0.0043	0.8619±0.0069	0.8391±0.0055	0.9832±0.0016	0.0000 ^†^

**Table 9:** Effect of ensemble size on macro-F1 across the three IoT intrusion-detection datasets. All ensemble configurations use the same data partitions, cross-validation folds, preprocessing, and hyperparameter-search settings as the models reported in Table 6, Table 7 and Table 8. Δ denotes the absolute change in macro-F1 relative to the preceding ensemble configuration within the same dataset.

Dataset	# Members	Ensemble Composition	Macro-F1	Δ Macro-F1
CICIoT2023	2	SoftVote-2: BagDT + LGBM	**0.8563 ± 0.0081**	-
	3	SoftVote-3: BagDT + LGBM + KNN	0.8315 ± 0.0073	−0.0248
	4	SoftVote-4: BagDT + LGBM + KNN + RF	0.8361 ± 0.0077	+0.0046
	3	SoftVote-Original: BagDT + KNN + GB	0.8085 ± 0.0074	-
TON_IoT	2	SoftVote-2: BagDT + LGBM	**0.9403 ± 0.0047**	-
	3	SoftVote-3: BagDT + LGBM + KNN	0.9392 ± 0.0058	−0.0011
	4	SoftVote-4: BagDT + LGBM + KNN + RF	0.9396 ± 0.0053	+0.0004
Edge-IIoTset	2	SoftVote-2: BagDT + LGBM	**0.8447 ± 0.0054**	-
	3	SoftVote-3: BagDT + LGBM + KNN	0.8403 ± 0.0052	−0.0044
	4	SoftVote-4: BagDT + LGBM + KNN + RF	0.8391 ± 0.0055	−0.0012

**Table 10:** Macro-F1 comparison between the strongest single learner and soft-voting ensembles of different sizes across the three datasets.

Dataset	LightGBM	SoftVote-2	SoftVote-3	SoftVote-4	Best Configuration
CICIoT2023	0.8506	**0.8563**	0.8315	0.8361	SoftVote-2
TON_IoT	**0.9411**	0.9403	0.9392	0.9396	LightGBM
Edge-IIoTset	**0.8482**	0.8447	0.8403	0.8391	LightGBM

**Table 11:** Marginal predictive benefit of the best soft-voting ensemble relative to LightGBM.

Dataset	LightGBM	Best Ensemble	Δ Macro-F1
CICIoT2023	0.8506	**0.8563**	+0.0057
TON_IoT	**0.9411**	0.9403	−0.0008
Edge-IIoTset	**0.8482**	0.8447	−0.0035

**Table 12:** Computational cost on CICIoT2023, reported as mean ± standard deviation over 20 fold estimates. Hardware: Intel Core i9-9900K at 3.60 GHz with 16 logical cores and 32 GB RAM. All models were trained on a CPU without GPU acceleration.

Model	Train (s)	Batch (ms/sample)	Single (ms)	Memory (MB)	Size (MB)
DT	32.5±1.2	0.001±0.000	0.9±0.0	71.7±3.6	74.2±0.0
BaggingDT	62.5±2.4	0.075±0.001	244.4±6.9	103.4±46.4	172.4±1.0
KNN	29.5±0.2	0.158±0.002	7.9±0.3	121.3±48.6	137.5±0.1
GB	770.9±21.2	0.008±0.000	1.5±0.0	70.3±3.1	74.0±0.0
RF	40.6±1.1	0.012±0.001	40.0±0.5	363.8±41.1	480.9±2.3
LightGBM	51.5±19.5	0.020±0.006	4.1±1.0	83.7±20.6	80.1±0.0
XGBoost	48.3±2.7	0.007±0.000	3.8±0.8	82.5±24.4	79.2±0.1
CatBoost	48.7±1.7	0.002±0.000	1.9±0.1	138.8±16.4	74.3±0.0
SoftVote-Original (BagDT + KNN + GB)	791.0±1.4	0.860±0.005	285.4±2.7	524.4±92.8	238.1±1.0
SoftVote-2 (BagDT + LGBM)	107.2±16.3	0.114±0.014	355.1±71.7	114.0±70.8	179.5±1.0
SoftVote-3 (BagDT + LGBM + KNN)	89.8±12.5	0.299±0.030	311.3±54.0	173.3±78.8	244.1±1.0
SoftVote-4 (BagDT + LGBM + KNN + RF)	80.8±0.5	0.268±0.004	308.8±3.4	466.3±134.5	652.1±2.9

**Table 13:** Quantitative evaluation of explanation quality.

Dataset	Stability Top-7 Jaccard	Surrogate R^2^	Δ*p* Top-*k*	Δ*p* Random	LIME-SHAP Jaccard@15
CICIoT2023	0.528±0.078	0.171	0.645	0.136	0.429
TON_IoT	0.214±0.023	0.200	0.463	0.066	0.111
Edge-IIoTset	0.364±0.062	0.208	0.708	0.127	0.304

**Table 14:** XAI-guided feature selection: performance retained against cost saved when the model is retrained on the fifteen highest-attribution features.

Dataset	Feature Set	Features	Accuracy	Macro-F1	F1 Retained (%)	Latency Saved (%)	Model Size (MB)
CICIoT2023	all features	46	0.9217	0.8591	100.0	0.0	197.8
CICIoT2023	XAI top-15	15	0.9127	0.8454	98.4	7.9	149.8
TON_IoT	all features	124	0.9730	0.9414	100.0	0.0	147.7
TON_IoT	XAI top-15	15	0.9709	0.9356	99.4	38.0	40.3
Edge-IIoTset	all features	59	0.8792	0.8515	100.0	0.0	108.7
Edge-IIoTset	XAI top-15	15	0.8787	0.8427	99.0	17.8	72.0

**Table 15:** The ten most important features globally on each dataset, ranked by mean absolute SHAP value. The ranking is based on the gradient-boosting component shared by the proposed ensemble and the single learner, which acts as the common global reference against which both models’ local explanations are assessed.

Rank	CICIoT2023	TON_IoT	Edge-IIoTset
1	IAT	src_ip_bytes	tcp.ack
2	Number	conn_state_REJ	http.request.version_0.0
3	rst_count	duration	http.request.method_0.0
4	Protocol Type	src_bytes	tcp.ack_raw
5	Variance	src_pkts	tcp.checksum
6	Weight	dst_ip_bytes	tcp.seq
7	Header_Length	dst_pkts	mqtt.conack.flags_0
8	Min	proto_tcp	tcp.flags
9	urg_count	dst_bytes	tcp.len
10	syn_count	conn_state_S0	icmp.seq_le

**Table 16:** Consistency between global (SHAP top-20) and local (LIME top-10) explanations, evaluated on one representative instance per class.

Dataset	Model	*N*	Overlap (%)	Range (%)	Surrogate $R^{2}$
CICIoT2023	Proposed	8	56.2±9.2	40–70	0.201
CICIoT2023	LightGBM	8	53.8±9.2	40–70	0.162
TON_IoT	Proposed	10	28.0±7.9	10–40	0.251
TON_IoT	LightGBM	10	25.0±10.8	10–40	0.144
Edge-IIoTset	Proposed	15	49.3±17.9	0–70	0.227
Edge-IIoTset	LightGBM	15	28.7±16.0	0–50	0.067

**Table 17:** LIME explanations for selected instances, showing the most and least influential features contributing to the classification decisions.

Instance	Most Influential Features	Least Influential Features	Predicted Class
71,452 (Recon)	ICMP, SSH, fin_flag_number	rst_flag_number, ARP, ack_count	Recon
1 (Web)	IAT, DNS, ICMP	ARP, rst_flag_number, ack_count	Web
6545 (Mirai)	Protocol Type, Magnitude	IAT, syn_flag_number, ack_count	Mirai

**Table 18:** Statistical significance testing at α=0.05.

Dataset	Test	Comparison	Mean Diff.	*p*	Sig.
CICIoT2023	Wilcoxon	SoftVote-Original (BagDT + KNN + GB) vs. LightGBM	−0.04210	0.00e+00	Yes
CICIoT2023	Wilcoxon	SoftVote-2 (BagDT + LGBM) vs. LightGBM	+0.00569	1.00e−05	Yes
CICIoT2023	Wilcoxon	SoftVote-3 (BagDT + LGBM + KNN) vs. LightGBM	−0.01908	0.00e+00	Yes
CICIoT2023	Wilcoxon	SoftVote-4 (BagDT + LGBM + KNN + RF) vs. LightGBM	−0.01451	0.00e+00	Yes
CICIoT2023	Friedman	12 models	-	0.00e+00	Yes
CICIoT2023	McNemar	SoftVote-2 (BagDT + LGBM) vs. LightGBM	+0.00280	1.89e−02	Yes
TON_IoT	Wilcoxon	SoftVote-2 (BagDT + LGBM) vs. LightGBM	−0.00079	1.92e−02	Yes
TON_IoT	Wilcoxon	SoftVote-3 (BagDT + LGBM + KNN) vs. LightGBM	−0.00187	1.69e−03	Yes
TON_IoT	Wilcoxon	SoftVote-4 (BagDT + LGBM + KNN + RF) vs. LightGBM	−0.00146	3.65e−03	Yes
TON_IoT	Friedman	10 models	-	0.00e+00	Yes
TON_IoT	McNemar	SoftVote-2 (BagDT + LGBM) vs. LightGBM	−0.00015	8.14e−01	No
Edge-IIoTset	Wilcoxon	SoftVote-2 (BagDT + LGBM) vs. LightGBM	−0.00350	8.25e−02	No
Edge-IIoTset	Wilcoxon	SoftVote-3 (BagDT + LGBM + KNN) vs. LightGBM	−0.00786	0.00e+00	Yes
Edge-IIoTset	Wilcoxon	SoftVote-4 (BagDT + LGBM + KNN + RF) vs. LightGBM	−0.00901	0.00e+00	Yes
Edge-IIoTset	Friedman	10 models	-	0.00e+00	Yes
Edge-IIoTset	McNemar	SoftVote-2 (BagDT + LGBM) vs. LightGBM	+0.00038	7.09e−01	No

## Data Availability

The dataset used in this study is the publicly available CICIoT2023 dataset, provided by the Canadian Institute for Cybersecurity (CIC), University of New Brunswick. It can be accessed at https://www.unb.ca/cic/datasets/iotdataset-2023.html (accessed on 12 May 2025) and is described in [10]. The TON_IoT dataset is provided by the Cyber Range Lab, UNSW Canberra, and is publicly available at https://research.unsw.edu.au/projects/toniot-datasets (accessed on accessed on 25 July 2026). The Edge-IIoTset dataset is publicly available through IEEE DataPort at https://ieee-dataport.org/documents/edge-iiotset-new-comprehensive-realistic-cyber-security-dataset-iot-and-iiot-applications (accessed on 25 July 2026) [41,42,43].
